# Midazolam for sedation before procedures in adults and children: a systematic review update

**DOI:** 10.1186/s13643-021-01617-5

**Published:** 2021-03-05

**Authors:** Aaron Conway, Kristina Chang, Sebastian Mafeld, Joanna Sutherland

**Affiliations:** 1 Peter Munk Cardiac Centre, University Health Network, 585 University Ave, Toronto, ON M5G 2N2 Canada; 2 Lawrence S. Bloomberg Faculty of Nursing, University of Toronto, Toronto, Canada; 3 School of Nursing, Queensland University of Technology (QUT), Brisbane, Australia; 4 Interventional Radiology, JDMI, Toronto General Hospital, Toronto, Canada; 5 Rural Clinical School, University of New South Wales, Coffs Harbour, NSW Australia; 6 Department of Anaesthesia, Coffs Harbour Health Campus, Coffs Harbour, NSW Australia

## Abstract

**Background:**

Midazolam is used for sedation before diagnostic and therapeutic medical procedures by several routes including oral, intravenous, intranasal and intramuscular. This is an update of a Cochrane review published in 2016, which aimed to determine the evidence on the effectiveness of midazolam for sedation when administered before a diagnostic or therapeutic procedure in adults and children.

**Methods:**

We searched CENTRAL, MEDLINE, Embase and two trials registers up to May 2020 together with reference checking to identify additional studies. We imposed no language restrictions. Randomized controlled trials of midazolam in comparison with placebo or other medications used for sedation were included. Two authors independently extracted data and assessed risk of bias for each included study.

**Results:**

Eight new trials were included in this update, which resulted in changed conclusions for the intravenous midazolam versus placebo, oral midazolam versus chloral hydrate and oral midazolam versus placebo comparisons. Effect estimates for all outcomes within the intravenous midazolam versus placebo (7 trials; 633 adults and 32 children) are uncertain due to concerns about imprecision and risk of bias. Midazolam resulted in a higher level of sedation than placebo (mean difference (MD) 1.05; 95% confidence interval (95% CI) 0.69 to 1.41; 1 study; 100 adults). There was no difference in anxiety (RR 0.43, 95% CI 0.09 to 1.99; *I*^2^ = 75%; 2 studies; 123 adults). Risk of difficulty performing procedures was lower in the midazolam group (RR 0.5; 95% CI 0.29 to 0.86; *I*^2^ = 45%; 3 studies; 191 adults and 32 children). There was no difference in discomfort (RR 0.51; 95% CI 0.25 to 1.04; *I*^2^ = 0%; 2 studies; 190 adults). Five trials with 336 children were included in the oral midazolam versus chloral hydrate comparison. Midazolam was less likely to result in moderate sedation (RR 0.30, 95% CI 0.11 to 0.82; *I*^2^ = 64%; 2 studies, 228 participants). This effect estimate is highly uncertain due to concerns about the risk of bias, imprecision and inconsistency. There was no difference in ratings of anxiety (SMD − 0.26; 95% CI − 0.75 to 0.23; *I*^2^ = 0%; 2 studies; 68 participants). Midazolam increased risk of incomplete procedures (RR 4.01; 95% CI 1.92 to 8.40; *I*^2^ = 0%; 4 studies, 268 participants). This effect estimate is uncertain due to concerns about the risk of bias. There were four trials with 359 adults and 77 children included in the oral midazolam versus placebo comparison. Midazolam reduced ratings of anxiety (SMD − 1.01; 95% CI − 1.86 to − 0.16; *I*^2^ = 92%; 4 studies; 436 participants). It is unclear if midazolam has an effect on difficulty performing procedures. Meta-analysis was not performed because there was only one incomplete procedure in the midazolam group in one of the trials. Midazolam reduced pain in one study with 99 adults (MD − 2; 95% CI − 2.5 to − 1.6; moderate quality). The effect estimate is uncertain due to concerns about the risk of bias.

**Conclusion:**

The additional evidence arising from inclusion of new studies in this updated review has not produced sufficient high-quality evidence to determine whether midazolam produces more effective sedation than other medications or placebo in any specific population included in this review. For adults, there was low-quality evidence that intravenous midazolam did not reduce the risk of anxiety or discomfort/pain in comparison to placebo, but the sedation level was higher. By combining results from adults and children, there was low-quality evidence of a large reduction in the risk of procedures being difficult to perform with midazolam in comparison to placebo. The effect estimates for this comparison are uncertain because there was concern about risk of bias and imprecision. There is moderate-quality evidence suggesting that oral midazolam produces less-effective sedation than chloral hydrate for completion of procedures for children undergoing non-invasive diagnostic procedures. Ratings of anxiety were not different between oral midazolam and chloral hydrate. The extent to which giving oral midazolam to adults or children decreases anxiety during procedures compared with placebo is uncertain due to concerns about risk of bias and imprecision. There was moderate-quality evidence from one study that oral midazolam reduced the severity of discomfort/pain for adults during a brief diagnostic procedure in comparison with placebo.

**Supplementary Information:**

The online version contains supplementary material available at 10.1186/s13643-021-01617-5.

## Background

Anxiety at the time of therapeutic or diagnostic medical procedures is a natural response to the unfamiliar environment and experience [1, 2]. Anxiety reduction (anxiolysis) may be achieved through pharmacological, and non-pharmacological means, with or without associated sedation [1, 3, 4]. Anxiolysis without conscious-level depression is termed minimal sedation [5]. If the medication induces an appreciable depression of conscious level (whilst the patient remains responsive), this is termed moderate sedation [5].

Midazolam is one of the most commonly used medications for inducing anxiolysis or sedation or both, prior to diagnostic and therapeutic procedures [6, 7]. This report is an update from a previous version of our Cochrane review [8]. Research interest in using midazolam for sedation before procedures persists, so it is important that new findings are incorporated into our review and disseminated. A comprehensive report of the methods was published with the original review. This report is restricted to highlighting the minor differences in methods which were applied between the previous version and this review, as well as describing the results and conclusions that have changed from the original version.

## Methods

A full description of the methods was provided in the original review [8], so we have not repeated them here and instead included them in Additional File 1. In brief, we searched the Cochrane Central Register of Controlled Trials (CENTRAL) (up to May 2020), MEDLINE in Ovid (1966 to May 2020) and Ovid Embase (1980 to May 2020). The search terms used to identify relevant trials in the original and updated review is presented in Additional File 2. Table 1 displays a summary of the inclusion and exclusion criteria. In the original review, we excluded the comparison between midazolam and dexmedetomidine because there was a Cochrane protocol focusing specifically on that comparison. That protocol has been abandoned. For this reason, we now included the dexmedetomidine comparison. The other difference in methods between the published Cochrane review and this update was the selection of primary outcomes. For this update, we refined the primary and secondary outcomes based on recommendations from the Sedation Consortium on Endpoints and Procedures for Treatment, Education, and Research Recommendations (SCEPTER) about core outcome domains in clinical trials of in procedural sedation, which were published after our initial review [9]. Recommended core outcome measures from SCEPTER included sedation level, proceduralist satisfaction and patient-centred outcomes, such as pain. Two authors independently performed screening and study selection as well as performed risk of bias assessments using the Cochrane ‘Risk of bias’ tool. Meta-analytic estimates for outcomes reported by two or more studies were calculated. As all types of procedures were included, it was possible that intervention effect could have varied across studies. For this reason, we expected a random-effects model would be most suitable. A fixed-effect model was considered when smaller values of the *I*^2^ statistic were first observed. There was an insufficient number of studies to perform subgroup analyses based on age, type of procedure and medical specialty or sensitivity analyses for trials rated low versus moderate or high risk of bias. We used the GRADE system to assess the quality of evidence and created summary of findings tables.

**Table 1:** Inclusion and exclusion criteria

Criteria	Description
*Studies*	• Randomized Controlled Trials in which midazolam was used for sedation before a procedure • No exclusions based on language or publication status
*Participants*	• Adults or children • Studies that included participants undergoing dental procedures were excluded
*Interventions*	• Studies that used midazolam by any route, at any dose or time, administered before a procedure • Studies that compared different drugs and different routes were excluded (e.g. intranasal midazolam plus intravenous sedative A versus intranasal sedative A plus intravenous midazolam; intravenous midazolam versus intranasal sedative A)
*Outcomes*	**Primary** • Level of sedation on a sedation assessment scale • Anxiety • Incomplete procedures/difficulty performing procedures • Discomfort/pain **Secondary** • Anterograde amnesia • Over-sedation • Disinhibition or excitation • Quality of recovery • Allergic or anaphylactoid reactions • Sedation reversal • Tolerance of procedure or participant cooperation • Participant or proceduralist satisfaction

## Results

### Included studies

**Fig. 1:**
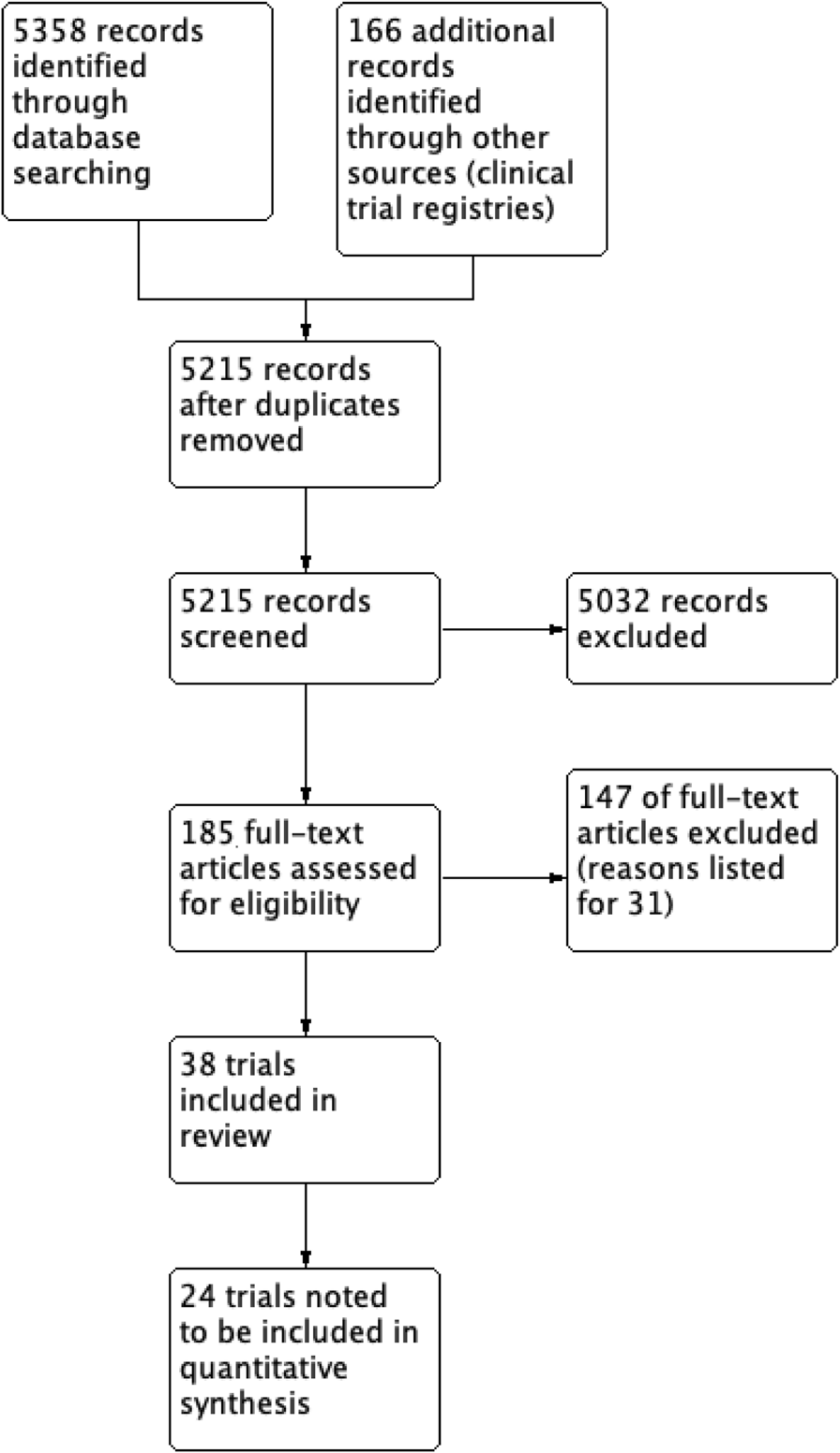
PRISMA flow diagram

**Fig. 2:**
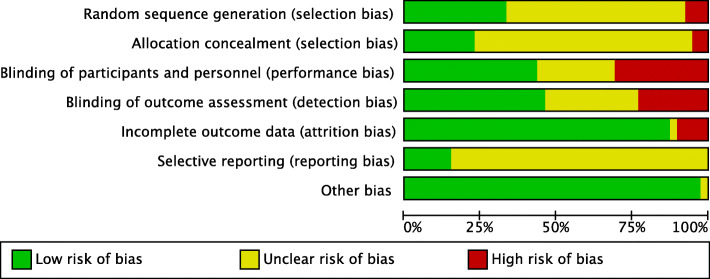
Risk of bias across studies

**Fig. 3:**
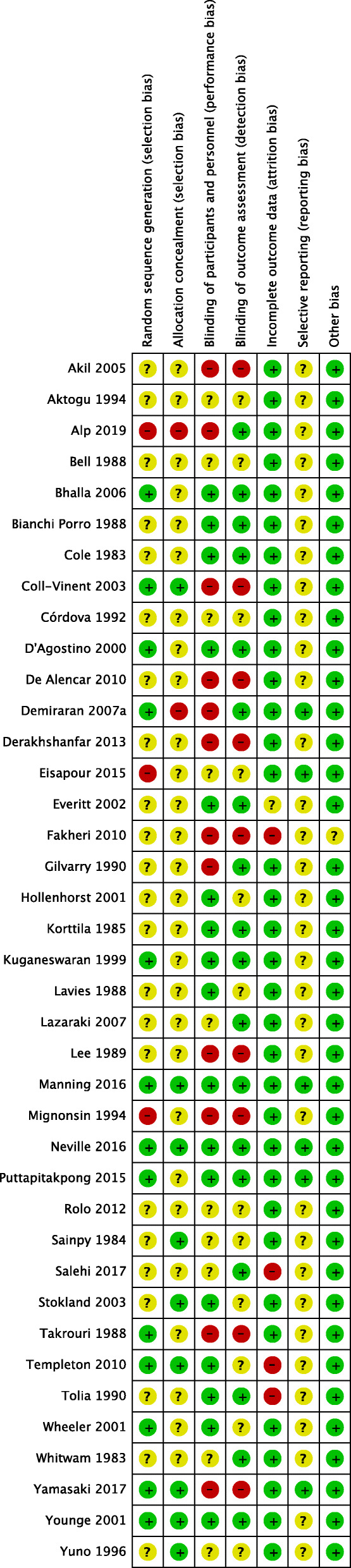
Risk of bias within studies

We included eight new trials in this updated review. In total, we included 38 trials with 3344 participants, which compared pre-procedure midazolam via the intravenous, oral and intranasal routes of administration, to either a placebo or alternative medication used for sedation (Fig. 1). The included trials were conducted in both adults and children. Summaries of the judgements of the risk of bias of included trials in Figs. 2 and 3. Details of the included trials are available in Additional File 3. The overall risk of performance bias and detection bias was low for 50% of the included trials. For randomization sequence generation and allocation concealment, the quality assessment yielded low risk of bias for approximately 25% or less of the included trials. The risk of attrition bias for the primary outcomes was low for more than 75% of trials. An expanded description of results for all comparisons is included in Additional File 4. Data and results of meta-analyses for all comparisons in the update are in Additional File 5. The remainder of this results section focuses on the comparisons with new evidence available in the update and new comparisons in this update.

### Comparisons with new evidence available in the update

#### Intravenous midazolam versus intravenous placebo

Intravenous midazolam was compared with placebo in six trials with 633 adult participants [10–15] and one trial with 32 children [16]. We downgraded the evidence to low quality on all four primary outcomes, due to concerns about the risk of bias and imprecision (Table 2).

**Table 2:** Intravenous midazolam compared to intravenous placebo for sedation before procedures

Patient or population: Adults requiring sedation before gastrointestinal endoscopy and bronchoscopy, adults requiring nasogastric tube insertion in an emergency department and children Settings: Hospitals in India, Iran, UK, Portugal, USA and Japan Intervention: Intravenous midazolam Comparison: Placebo						
**Outcomes**	**Illustrative comparative risks**^a^ **(95% CI)**		**Relative effect** **(95% CI)**	**No of participants** **(studies)**	**Quality of the evidence** **(GRADE)**	**Comments**
	**Assumed risk**	**Corresponding risk**				
	**Placebo**	**Intravenous midazolam**				
**Level of sedation on a sedation assessment scale** The Ramsay scale was used (numerical scale that ranged from 1 to 6 with higher scores indicating the participant was more sedated)	1.19	1 Higher (from 0.6 higher to 1.4 higher		100 (1 study)	**Low**^1^ ⊕⊕⊝⊝	
**Numeric rating of anxiety or number of participants rated as anxious** Number of participants rated as anxious	**333 per 1000**	**143 per 1000** (30 to 663)	**RR 0.43** (0.09 to 1.99)	123 (2 studies)	**low**^2^ ⊕⊕⊝⊝	
**Proportion of incomplete procedures or where there was difficulty performing the procedures**	**216 per 1000**	**108 per 1000** (63 to 186)	**RR 0.50** (0.29 to 0.86)	223 (3 studies)	**Low**^3^ ⊕⊕⊝⊝	
**Discomfort/pain**	**168 per 1000**	**86 per 1000** (42 to 175)	**RR 0.51** (0.25 to 1.04)	190 (2 studies)	**Low**^4^ ⊕⊕⊝⊝	

^a^The basis for the **assumed risk** is the control group risk across studies or the average risk for pooled data and the control group risk for single studies. The **corresponding risk** (and its 95% confidence interval) is based on the assumed risk in the comparison group and the **relative effect** of the intervention (and its 95% CI)

***CI*** Confidence interval; ***RR*** Risk ratio

GRADE Working Group grades of evidence

**High quality:** Further research is very unlikely to change our confidence in the estimate of effect.

**Moderate quality:** Further research is likely to have an important impact on our confidence in the estimate of effect and may change the estimate.

**Low quality:** Further research is very likely to have an important impact on our confidence in the estimate of effect and is likely to change the estimate.

**Very low quality:** We are very uncertain about the estimate.

*Footnotes*

^1^Downgraded two levels due to concerns about the risk of bias (it was unclear how the allocation sequence was generated and concealed and how participants were blinded to the allocation) and imprecision (optimal information size was not met—single study with a small number of participants, no confidence intervals were reported)

^2^Downgraded two levels due to concerns about risk of bias (it was unclear in one study how the allocation sequence was generated and concealed and how participants were blinded to the allocation) and imprecision (optimal information size was not met—single study with a small number of participants, wide confidence intervals crossing the line of no effect, and including the potential for both benefit and harm)

^3^Downgraded two levels due to concerns about the risk of bias (it was unclear in one study how the allocation sequence was concealed) and imprecision (optimal information size was not met—wide confidence intervals including the potential for a very large benefit or very small degree of harm)

^4^Downgraded two levels due to concerns about the risk of bias (it was unclear in one study how the allocation sequence was concealed) and imprecision (optimal information size was not met—wide confidence intervals including the potential for a very large benefit or very small degree of harm)

##### Primary outcomes

***Level of sedation on a sedation assessment scale***

One study, which used the Ramsay scale to measure level of sedation, reported on this outcome [14]. Scale scores range from 1 to 6, with higher scores indicating the participant was more sedated. Participants randomized to midazolam were more sedated (mean difference (MD) 1.05; 95% confidence interval (95% CI) 0.6 to 1.4; 1 study; 100 participants; low quality). The quality of this evidence was downgraded to low quality due to concerns about risk of bias and imprecision.

***Numeric rating scale of anxiety or number of participants rated as anxious***

There was no difference in anxiety (risk ratio (RR) 0.43; 95% CI 0.09 to 1.99; *I*^2^ = 75%; 123 adults; 2 studies; low quality). The quality of this evidence was downgraded to low quality due to concerns about risk of bias and imprecision.

***Proportion of incomplete procedures or where there was difficulty performing the procedures***

Risk of difficulty performing procedures was lower in the midazolam group (RR 0.5; 95% CI 0.29 to 0.86; *I*^2^ = 45%; 3 studies; 191 adults and 32 children; low quality). The quality of this evidence was downgraded to low quality due to concerns about risk of bias and imprecision.

***Discomfort/pain***

There was no difference in discomfort between groups (RR 0.51; 95% CI 0.25 to 1.04; *I*^2^ = 0%; 2 studies; 190 participants; low-quality). The quality of this evidence was downgraded to low quality due to concerns about risk of bias and imprecision.

##### Secondary outcomes

No trials reported results for disinhibition or excitation, quality of recovery, allergy or anaphylactoid reactions and tolerance of procedure or patient cooperation. For anterograde amnesia (defined by the number of participants who recalled the procedure), there was no difference between groups in one study (RR 0.83; 95% CI 0.52 to 1.32; 1 study; 23 participants; low-quality evidence) [13].

***Sedation reversal***

One trial (100 participants) reported that no participants required sedation reversal in either group [14].

***Participant or proceduralist satisfaction***

Four trials, all conducted with adult participants, reported on participant or proceduralist satisfaction (Bhalla 2006; Manning 2016; Rolo 2012; Yuno 1996) [10, 13–15]. Midazolam increased the number of participants who were satisfied with sedation (RR 1.21; 95% CI 1.07 to 1.36; trials = 2; participants = 123; *I*^2^ = 0%; moderate quality). In the Yuno 1996 trial, participant satisfaction, which was measured on a four-point scale with lower scores indicating greater satisfaction, was better in the midazolam group (MD − 1.65; 95% CI − 1.75 to − 1.55; 40 participants; moderate quality). Proceduralist satisfaction was also greater in the midazolam group in the same study (MD − 1.8; 95% CI − 1.9 to − 1.7; 1 study; 40 participants; moderate quality). The effect estimates for this outcome are uncertain due to concerns about the risk of bias.

#### Oral midazolam versus chloral hydrate

Five trials (Akil 2005; D’Agostino 2000; Derakhshanfar 2013; Salehi 2017; Wheeler 2001), with 336 participants compared oral midazolam with chloral hydrate for sedation of children [17–21] (Table 3).

**Table 3:** Oral midazolam compared to oral chloral hydrate for sedation before procedures

Patient or population: Children requiring sedation before procedures that require motion control, including echocardiography, lumbar puncture, micturating cystourethrograms, and neuroimaging Settings: Paediatric ICU in USA, emergency departments in USA and Iran and Medical Imaging department in Turkey Intervention: Oral midazolam Comparison: Oral chloral hydrate						
**Outcomes**	**Illustrative comparative risks**^a^ **(95% CI)**		**Relative effect** **(95% CI)**	**No of participants** **(studies)**	**Quality of the evidence** **(GRADE)**	**Comments**
	**Assumed risk**	**Corresponding risk**				
	**Chloral hydrate**	**Oral midazolam**				
**Level of sedation on sedation assessment scale** Derakhshanfar 2013 reported the number of patients reaching moderate sedation on Wheeler’s sedation scale and Salehi 2017 reported the number of patients reaching moderate sedation on the RASS scale.	**596 per 1000**	**179 per 1000** (66 to 489)	**RR 0.3** (0.11 to 0.82)	228 (2)	**Very low**^1^ ⊕⊝⊝⊝	
**Numeric rating of anxiety or number of participants rated as anxious** (numerical rating scale of 1–5 with lower scores indicating less anxiety)	2.5	MD was **0.77 lower**^2^ (2.2 lower to 0.68 higher)		88 (2)	**Low**^3^ ⊕⊕⊝⊝	The assumed and corresponding risks were estimated from the SMD, which was − 0.26 (95% CI − 0.75 to 0.23).
**Proportion of incomplete procedures or where there was difficulty performing the procedures**	**56 per 1000**	**226 per 1000** (108 to 474)	**RR 4.01** (1.92 to 8.4)	268 (4)	**Moderate**^4^ ⊕⊕⊕⊝	
**Discomfort/pain** (as defined/measured by the authors of the trial)						No studies reported on this outcome.

^a^The basis for the **assumed risk** is the control group risk across studies or the average risk for pooled data and the control group risk for single studies. The **corresponding risk** (and its 95% confidence interval) is based on the assumed risk in the comparison group and the **relative effect** of the intervention (and its 95% CI).

***CI*** Confidence interval; ***RR*** Risk ratio

GRADE Working Group grades of evidence

**High quality:** Further research is very unlikely to change our confidence in the estimate of effect.

**Moderate quality:** Further research is likely to have an important impact on our confidence in the estimate of effect and may change the estimate.

**Low quality:** Further research is very likely to have an important impact on our confidence in the estimate of effect and is likely to change the estimate.

**Very low quality:** We are very uncertain about the estimate.

*Footnotes*

^1^Downgraded three levels due to concerns about risk of bias (both studies had unclear and high risk of bias for multiple domains), inconsistency (although the effect estimates for both studies indicated midazolam was less likely to result in moderate sedation, the *I*^2^ value was high) and imprecision (wide confidence intervals indicating the effect could be either very large or small)

^2^Studies in this comparison used different instruments to measure anxiety. We used the SMD for meta-analysis. We selected the D’Agostino 2000 trial as our representative study in order to calculate the assumed risk and corresponding risk for the summary of findings table. The standard deviation for the placebo group in this study was 2.97, measured on a scale ranging from 1 to 5

^3^Downgraded three levels due to concerns about risk of bias (both studies had unclear and high risk of bias for multiple domains) and imprecision (optimal information size was not met—only a small number of participants, wide confidence intervals crossing the line of no effect, and including the potential for both benefit and harm)

^4^Downgraded one level due to concerns about risk of bias (all studies had unclear and high risk of bias for multiple domains)

##### Primary outcomes

***Level of sedation on a sedation assessment scale***

Two trials reported on the rate of reaching a level of moderate sedation. Derakhshanfar et al. [17] reported the number of patients reaching moderate sedation on Wheeler’s sedation scale, and Salehi et al. [18] reported the number of patients reaching moderate sedation on the RASS scale. Different scales were used to measure the level of sedation in these studies. Derakhshanfar et al. [17] used Wheeler’s sedation level with scores ranging from 1 = agitated to 4 = eyes closing spontaneously but with a response to minor stimuli. Salehi et al. [18] reported using the RASS, with the levels of ‘alert and calm’, ‘drowsy’, ‘light sedation’ and ‘moderate sedation’. Based on guidelines from the American Society of Anesthesiology, the category in the Wheeler scale that corresponds most closely to ‘moderate sedation’ was level 4 (eyes closing spontaneously but with a response to minor stimuli) (American Society of Anesthesiologists 2014). We used this definition for the meta-analysis to combine results from the two studies. Meta-analysis of results suggested that midazolam was less likely to result in moderate sedation compared with chloral (RR 0.30; 95% CI 0.11 to 0.82; *I*^2^ = 64%; 2 studies; 228 participants; very low-quality). We downgraded the evidence from this meta-analysis to very low quality, due to concerns about the risk of bias, inconsistency and imprecision.

***Numeric rating scale of anxiety or number of participants rated as anxious***

A numerical rating of anxiety was reported in two trials with 88 participants. The outcome was measured using different scales (by children using a numerical rating scale in D’Agostino et al. [19] and by parents using the Spielberger’s Trait Anxiety Inventory in Akil et al. [20]). The standardized mean difference in anxiety rating was not different (standardized mean difference (SMD) − 0.26; 95% CI − 0.75 to 0.23; *I*^2^ = 0%; 2 studies; 68 participants; low quality). We downgraded the evidence for this outcome to low, due to concerns about the risk of bias and imprecision. To aid interpretation, we converted the estimate for the SMD to an MD using the numerical rating scale in D’Agostino et al. [19]. Scores ranged from 1 to 5 with lower scores indicating less anxiety). The standard deviation for the placebo group in this study was 2.97. The mean difference for the meta-analysis was − 0.7 (95% CI − 2.2 to 0.7).

***Proportion of incomplete procedures or where there was difficulty performing the procedures***

Four trials (268 participants) reported on this outcome [17, 19–21]. Incomplete procedures were more likely in the midazolam group (RR 4.01; 95% CI 1.92 to 8.40; *I*^2^ = 0%; 4 studies; 436 participants; moderate quality). We downgraded the quality of evidence to moderate, due to concerns about the risk of bias.

***Discomfort/pain***

No trials reported this outcome.

##### Secondary outcomes

Within this comparison, no trials reported results for anterograde amnesia, quality of recovery, allergic or anaphylactoid reactions, sedation reversal and patient or proceduralist satisfaction.

***Disinhibition or excitation***

There was no difference in disinhibition or excitation between midazolam or chloral hydrate groups in the Derakhshanfar et al. [17] trial (RR 1.0; 95% CI 0.39 to 2.55; 1 study; 160 participants). No events were observed in either group by Wheeler et al. [21] (40 participants). Quality of evidence was downgraded to low quality due to concerns about risk of bias and imprecision.

***Tolerance of procedure or participant cooperation***

Tolerance of the procedure was measured using the Frankl behaviour rating scale (range 1 to 4, with higher scores indicating better tolerance) by Akil et al. [20]. There was no difference in tolerance between groups (MD 0.25; 95% CI − 0.9 to 0.4; 1 study; 35 participants; low quality). Participant cooperation was measured using the Houpt behavioural scale (range 1 to 6, with higher scores indicating better cooperation) in the Akil et al. [20] trial, and there was no difference between groups (MD 0.16; 95% CI − 0.54 to 0.86; 1 study; 35 participants; low quality). The evidence for this outcome was rated as low quality due to concerns about risk of bias and imprecision.

#### Oral midazolam versus placebo

Four trials (Akil 2005; Kuganeswaran 1999; Puttapitakpong 2015; Templeton 2010) with 436 participants compared midazolam administered via the oral route with a placebo (Table 4) [20, 22–24]. Two trials were conducted with adults and two with children.

**Table 4:** Oral midazolam compared to oral placebo for sedation before procedures

Patient or population: Children requiring sedation before micturating cystourethrograms, and Kirschner wire removal, and adults undergoing endoscopy Settings: X-ray department in Turkey, orthopaedic outpatient department in UK, and endoscopy suites in USA and Thailand Intervention: Oral midazolam Comparison: Placebo						
**Outcomes**	**Illustrative comparative risks**^a^ **(95% CI)**		**Relative effect** **(95% CI)**	**No of participants** **(studies)**	**Quality of the evidence** **(GRADE)**	**Comments**
	**Assumed risk**	**Corresponding risk**				
	**Placebo**	**Midazolam**				
**Level of sedation on a sedation assessment scale** (as defined/measured by the authors of the trial)						No studies reported on this outcome.
**Numeric rating of anxiety or number of participants rated as anxious** (as defined/measured by the authors of the trial)	4.6^2^ (measured on a scale that ranged from 0 to 10 with higher scores representing worse anxiety)	MD was **1.9 lower** (3.5 lower to 0.3 lower)		436(4)	**Low**^1^ ⊕⊕⊝⊝	The assumed and corresponding risks were estimated from the SMD, which was − 1 (95% CI − 1.86 to − 0.16).
**Proportion of incomplete procedures or where there was difficulty performing the procedures** (as defined/measured by the authors of the trial)				439 (4 studies)	**Low**^1^ ⊕⊕⊝⊝	Relative effect was not able to be conducted because there was only one incomplete procedure in the midazolam group in one of the four trials that reported on this outcome.
**Discomfort/pain** (**as defined/measured by the authors of the trial)** Scores ranged from 0 to 10 with higher score indicating more pain	4.62	MD was **2 lower** (2.5 lower to 1.6 lower)		99 (1 study)	**Moderate**^1^ ⊕⊕⊕⊝	

^a^The basis for the **assumed risk** is the control group risk across studies or the average risk for pooled data and the control group risk for single studies. The **corresponding risk** (and its 95% confidence interval) is based on the assumed risk in the comparison group and the **relative effect** of the intervention (and its 95% CI).

***CI*** Confidence interval; ***RR*** Risk ratio

GRADE Working Group grades of evidence

**High quality:** Further research is very unlikely to change our confidence in the estimate of effect.

**Moderate quality:** Further research is likely to have an important impact on our confidence in the estimate of effect and may change the estimate.

**Low quality:** Further research is very likely to have an important impact on our confidence in the estimate of effect and is likely to change the estimate.

**Very low quality:** We are very uncertain about the estimate.

*Footnotes*

^1^Downgraded two levels due to concerns about the risk of bias and imprecision

^2^Studies in this comparison used different instruments to measure anxiety. We used the SMD for meta-analysis. We selected the Puttapitakpong 2015 trial as our representative study in order to calculate the assumed risk and corresponding risk for the summary of findings table. The standard deviation for the placebo group in this study was 1.9, measured on a scale ranging from 0 to 10

^2^Downgraded one level due to concerns about the risk of bias

##### Primary outcomes

***Level of sedation on a sedation assessment scale***

Kuganeswaran et al. [22] reported on level of sedation measured on a 4-point scale with higher scores indicating a greater sedative effect. Although it was reported that level of sedation was measured every 5 min, summary statistics were reported only for the timepoint 10 min after administration of midazolam. At this timepoint, sedation level was higher in the midazolam group (MD 0.2; 95% CI 0.19 to 0.21; 101 participants; low-quality evidence). Numeric rating scale of anxiety or number of participants rated as anxious A numerical rating of anxiety was reported in all trials included in this comparison. Standardized mean difference was used for meta-analysis because different scales were used in each trial. Midazolam reduced ratings of anxiety by one standard deviation (SMD − 1.01; 95% CI − 1.86 to − 0.16; *I*^2^ = 92%; 4 studies; 436 participants; low quality). The quality of this evidence was downgraded to low quality due to concerns about the risk of bias and imprecision. To aid interpretation, we converted the estimate for the SMD to an MD using the numerical rating scale from Puttapitakpong et al. [23]. Scores ranged from 0 to 10, with lower scores indicating less anxiety. The standard deviation for the placebo group in this study was 1.9. The mean difference for the meta-analysis was − 1.9 (95% CI = − 3.5 to 0.3).

***Proportion of incomplete procedures or where there was difficulty performing the procedures***

There were no incomplete procedures in either the midazolam or placebo groups in three trials [22–24]. One procedure (6%) could not be completed in the midazolam group in Akil et al. [20].

***Discomfort/pain***

In the Kuganeswaran et al. [22] trial, which was conducted with adult participants undergoing sigmoidoscopy, pain was lower in the midazolam group (MD − 2; 95% CI − 2.5 to − 1.6; 1 study; 99 participants; moderate quality). Quality of evidence was downgraded due to concerns about the risk of bias.

##### Secondary outcomes

Within this comparison, no trials reported results for disinhibition or excitation, quality of recovery, allergic or anaphylactoid reactions and sedation reversal. For anterograde amnesia (defined by number of participants who recalled the procedure), there was no overall difference between midazolam and placebo in meta-analysis of two trials that enrolled adults undergoing upper (Puttapitakpong et al. [23]) or lower (Kuganeswaran et al. [22]) endoscopy (RR 0.32, 95% CI 0.01 to 10.12; *I*^2^ = 99%; 2 trials; 359 participants; low quality). However, the results were inconsistent and imprecise. As such, the quality of evidence was rated as low quality.

***Tolerance of procedure or participant cooperation***

Tolerance of the procedure was measured using the Frankl behaviour rating scale (range 1 to 4, with higher scores indicating better tolerance) in Akil et al. [20]. There was no difference in tolerance between groups (MD − 0.13, 95% CI − 0.5 to 0.76; 1 study; 35 participants; low quality). This effect estimate is uncertain due to concerns about imprecision and the risk of bias. Tolerance of the procedure (defined as not willing to repeat the procedure with the same sedation) was better in the midazolam group in the Puttapitakpong et al. [23] trial. Fewer participants in the midazolam group were not willing to repeat the procedure with the same sedation (RR 0.1 95% CI 0.01 to 0.77; 1 study; 260 participants; low-quality). This effect estimate is uncertain due to concerns about imprecision and the risk of bias.

Participant cooperation was measured using the Houpt behavioural scale (range 1 to 6, with higher scores indicating better cooperation) in Akil et al. [20]. Participant cooperation between groups was higher in the midazolam group, but the effect estimate was imprecise, and there were concerns about the risk of bias (MD 0.82, 95% CI 0.1 to 1.54; 1 study; 35 participants; low-quality).

***Participant or proceduralist satisfaction***

Participant satisfaction (measured by participants’ perception that they received inadequate sedation for their procedure) in Kuganeswaran et al. [22] was superior in the midazolam group (RR 0.43 95% CI 0.26 to 0.7; 1 study; 99 participants; low quality). This effect estimate is uncertain due to concerns about imprecision and the risk of bias. In the Puttapitakpong et al. [23] trial, ratings of satisfaction on a scale from 0 to 10 (higher scores = greater satisfaction) from participants (MD 2.5, 95% CI 2.18 to 2.82; 1 study; 260 participants; moderate quality) and proceduralists (MD 2.3 95% CI 2.02 to 2.58; 1 study; 260 participants; moderate quality due to concerns about the risk of bias) were higher in the midazolam group. The effect estimates from this trial are uncertain due to concerns about the risk of bias.

#### New comparisons in this update

##### Intranasal midazolam versus dexmedetomidine

One trial with 38 participants compared intranasal midazolam with intranasal dexmedetomidine for sedation in children before laceration repair [25]. Eighteen participants were randomized to receive 0.4mg/kg of intranasal midazolam, and twenty participants received 2 mcg/kg of intranasal dexmedetomidine. Within this comparison, no trials reported results for the level of sedation, incomplete or difficulty performing procedures, discomfort/pain and any of the secondary outcomes.

Participants’ level of anxiety during patient positioning for the procedure was measured in this trial by the modified Yale Preoperative Anxiety Scale [25]. Participants were observed for five categories (activity, vocalizations, emotional expressivity, state of apparent arousal, and use of parents) combined to produce a total anxiety score between 23.3 and 100, where higher values indicated greater anxiety. The dexmedetomidine group had a median anxiety score that was significantly lower compared to the midazolam group (23.3 (IQR 23–35) dexmedetomidine; 36.3 (IQR 33–41) midazolam), with a difference in score of 9.2 points (95% CI 5.0 to 13.3; *P* = 0.007). The proportion of participants who were not anxious during positioning for the procedure was also reported. Participants who scored less than 30 using the modified Yale Preoperative Anxiety Scale were considered ‘not anxious’. More participants in the dexmedetomidine group were not anxious during positioning compared to those in the midazolam group (14/20 (70%) dexmedetomidine; 2/18 (11%) midazolam, *P* = 0.00). The odds of participants not being anxious during positioning were 19 times higher in the dexmedetomidine group compared to the midazolam group (OR 19, 95% CI 3 to 108). We rated this evidence as moderate quality, due to concerns about imprecision.

##### Intranasal midazolam versus ketamine

One trial, with 145 children undergoing echocardiography, compared intranasal midazolam with ketamine [26]. There were 73 participants allocated to receive midazolam (0.2mg/kg) and 27 participants to ketamine (4mg/kg). Within this comparison, no trials reported results for anxiety, discomfort/pain and any of the secondary outcomes.

Level of sedation was measured every 15 min using the RASS, with levels of ‘awake and calm’, ‘drowsy’ or ‘sedated’. More participants were rated as ‘sedated’ in the midazolam group at 15 min (RR 50; 95% CI 3 to 809; 1 trial; 145 participants; low quality) and 30 min (RR 2; 95% CI 1.3 to 3.3; 1 trial; 145 participants; low quality). There was no difference in the level of sedation between groups at 45 min (RR 0.97; 95% CI 0.88 to 1.67; 1 trial; 145 participants; low quality) and 60 min (RR 1.0; 95% CI 0.97 to 1.03; 1 trial; 145 participants; low quality). The effect estimates for this outcome are uncertain due to concerns about imprecision and the risk of bias.

There was no difference between groups in the number of participants who were insufficiently sedated to be able to perform the procedure (RR 0.99; 95% CI 0.21 to 4.73; 1 trial; 145 participants; low quality). This effect estimate is uncertain due to concerns about imprecision and the risk of bias.

##### Intravenous midazolam versus pethidine hydrochloride

We identified one trial that enrolled 120 participants for this comparison [27]. Forty participants were randomized to midazolam and 39 to pethidine hydrochloride. Participants in the midazolam group received intravenous midazolam in 0.5–1.0 mg doses administered until a Ramsay score of 3 was achieved for pharyngeal observation. Participants in the pethidine group received 35 mg of intravenous pethidine hydrochloride. Within this comparison, no trials reported results for level of sedation, anxiety, incomplete or difficulty performing procedures, anterograde amnesia, disinhibition or excitation, quality of recovery, allergic or anaphylactoid reactions, sedation reversal, tolerance of procedure or patient cooperation and participant or proceduralist satisfaction.

Yamasaki et al. [27] reported on participants’ discomfort during pharyngeal observation using a visual analogue scale. Participants rated their level of discomfort between 0 and 100 mm along a 100-mm horizontal line, where higher values indicated greater pain. The mean score for discomfort was not significantly different between the midazolam and pethidine groups (MD − 0.4; 95% CI − 1.39 to 0.59; 1 study; 120 participants; low quality). This effect estimate is uncertain due to concerns about imprecision and the risk of bias.

## Discussion

### Summary of new evidence for comparisons included in the original review

Despite inclusion of additional studies in this update, in general, it remains unclear if intravenous midazolam is more effective than placebo for procedural sedation. Overall, we judged the quality of the evidence for the primary outcomes to be low quality. Intravenous midazolam did not reduce the risk of anxiety or discomfort/pain. By combining results from adults and children, there was low-quality evidence of a large reduction in the risk of procedures being difficult to perform with midazolam in comparison to placebo. Also, there was low-quality evidence from multiple studies that midazolam improved participant satisfaction in comparison with placebo. Further studies are needed to increase precision and consequently increase confidence in the effect estimates.

Based on a meta-analysis of four trials [17, 19–21], with 268 participants, midazolam was associated with a greater quantity of incomplete procedures in children when compared to chloral hydrate (RR 4.01, 95% CI 1.92 to 8.40). We rated the quality of the evidence as moderate (summary of findings, Table 2). This result is similar to another recently published meta-analysis with different inclusion criteria [28]. However, chloral hydrate was not associated with advantages in any other domain investigated in our review, including the level of pain and level of anxiety. It should be noted that chloral hydrate has an inconsistent duration of action and is unavailable in many regions, including the USA [29].

There was low-quality evidence that oral midazolam reduced anxiety in comparison with placebo in adults and children. There was low quality evidence of a reduction in discomfort/pain in one of the 4 studies included in this comparison [23].

### Evidence from new comparisons

There was moderate-quality evidence that children who received midazolam for laceration repair had higher ratings of anxiety compared with dexmedetomidine. Additional trials should be conducted in other similar clinical contexts where motion control is required. Such research is needed to confirm these promising initial findings indicating the potential superiority of intranasal dexmedetomidine over midazolam for this indication. Alongside these trials should be a consideration of the cost-effectiveness of dexmedetomidine in comparison to midazolam for paediatric sedation. Another new comparison was intranasal midazolam versus ketamine. One study was identified with 155 children undergoing echocardiography [26]. Low-quality evidence indicated that sedation level was higher in the midazolam group earlier after administration, but there was no difference in the number of procedures that could not be completed between groups.

### Limitations

Trial protocols were not sought for clarifications regarding risk of bias assessments because many included trials in this review were published prior to the establishment of clinical trial registries. For this update, we based our selection of primary outcomes on recommendations from the Sedation Consortium on Endpoints and Procedures for Treatment, Education, and Research Recommendations (SCEPTER) about core outcome domains in clinical trials of in procedural sedation [9]. The secondary outcomes we chose to exclude from this update for the review based on these recommendations were (1) vital signs, based on the fact that they are surrogate outcomes that are likely only important if they lead to clinical outcomes, (2) outcomes related to sedation onset and offset (being duration of sedation, onset of section and offset of sedation), and (3) over-sedation, because this outcome would be more objectively measured by the requirement for sedation reversal which is also an outcome in this review. These decisions about the handling of the data, which we made after seeing it, may have introduced bias to the review process. An additional limitation is that we were unable to conduct the planned subgroup analyses. In particular, elderly patients may be particularly sensitive to the sedative effects of midazolam, so it is unfortunate we were unable to conduct this specific subgroup analysis. It should also be noted that an inherent difficulty in evidence syntheses for medications used in procedural sedation is that all procedures differ in intensity and frequency of stimulation, which potentially impacts sedation efficacy. Our rationale for pooling results of studies that used different doses of midazolam, and also different procedures, was that, presumably, an appropriate dose of midazolam would have been chosen based on the intensity and frequency of stimulation for the procedures. That said, factors such as the dosage used and type of procedure performed could be reasons for the inconsistency in results between studies. An alternative approach that could be considered for similar systematic reviews in the future (or updates for this review) would be to only pool results from studies that used the same dosage of midazolam for the same procedures. Finally, this review was limited to studies that used a randomized controlled trial design. Consideration for the inclusion of studies that used non-randomized designs may be worthwhile for evidence syntheses on the effectiveness of midazolam for sedation before procedures in the future because the total number of studies included in each comparison was relatively small.

### Conclusion

The additional evidence arising from inclusion of new studies in this updated review has not produced sufficient high-quality evidence to determine whether midazolam produces more effective sedation than other medications in any specific population included in this review. Moderate-quality evidence demonstrated that midazolam administered orally to children who require sedation for motion control during diagnostic procedures produced less-effective sedation compared with chloral hydrate in terms of the ability to complete procedures. Patients appear to prefer to be sedated with midazolam when undergoing a procedure than receive no sedation at all. For this reason, sedation with midazolam could be offered if it is clinically appropriate to do so.

## Supplementary Information

**Additional file 1.** Methods: Expanded description of methods used in the original and updated review

**Additional file 2.** Search strategy: Search terms used for the original and updated review

**Additional file 3.** Study characteristics: Sample and intervention characteristics and outcome descriptions as well as risk of bias assessments for each study included in the updated review

**Additional file 4.** Results: Expanded description of results for all comparisons included in the updated review

**Additional file 5.** Data and analyses: All data and results of meta-analyses

## Data Availability

All data generated or analysed during this study are included in this published article (and its supplementary information files).

## Abbreviations

RR: Relative risk ratio
95% CI: 95% Confidence interval
MD: Mean difference
SMD: Standardized mean difference
