# Supplementary material for: Midazolam for sedation before procedures in adults and children: a systematic review update
Source: Syst Rev. 2021 Mar 5;10:69. doi: 10.1186/s13643-021-01617-5 (PMC7936483; doi:10.1186/s13643-021-01617-5)
Supplement: Supplementary file 1 — Additional file 1. Methods: Expanded description of methods used in the original and updated review [file 13643_2021_1617_MOESM1_ESM.docx]

# Methods

## Criteria for considering studies for this review

### Types of studies

We included randomized controlled trials (RCTs) in which midazolam was used for sedation before a procedure (diagnostic or therapeutic). We included studies irrespective of language and publication status. We excluded prospective cohort studies and quasi-randomized studies.

### Types of participants

We included participants of any age (adults and children) who were undergoing a procedure preceded by sedation. We excluded any participants undergoing dental procedures, because a Cochrane review has been published about sedation with midazolam specifically in that setting ([Ashley 2018](#REF-Ashley-2018)). No other restrictions were applied.

### Types of interventions

We included midazolam by any route, at any dose or time, administered before a procedure — participants who received a placebo before a procedure constituted the control group. We also included studies that compared midazolam with another drug for sedation before a procedure. We performed a separate analysis for each different drug comparison (for example, midazolam versus sedative A; midazolam versus sedative B). We selected three primary comparisons in the review:

- Intravenous midazolam versus placebo
- Oral midazolam versus chloral hydrate
- Oral midazolam versus placebo

These comparisons were designated as primary because evidence from multiple trials was available, including recent publications suggesting relevance to contemporary practice.

We excluded studies that simultaneously compared different drugs and different routes (for example, intranasal midazolam plus intravenous sedative A versus intranasal sedative A plus intravenous midazolam; intravenous midazolam versus intranasal sedative A).

In the previous version of this review, we excluded the comparison between midazolam and dexmedetomidine because there was a Cochrane protocol focusing specifically on this comparison. That protocol has been abandoned. For this reason, we now include the dexmedetomidine comparison (see [Differences between protocol and review](#PRO_REV_DIFF)).

### Types of outcome measures

#### Primary outcomes

For this update, we based our selection of primary outcomes on recommendations from the Sedation Consortium on Endpoints and Procedures for Treatment, Education, and Research Recommendations (SCEPTER) about core outcome domains in clinical trials of in procedural sedation. ([Williams 2017](#REF-Williams-2017)). Recommended core outcomes measures from SCEPTER included sedation level, proceduralist satisfaction and patient-centred outcomes, such as pain. Based on these recommendations, we considered it appropriate to designate the following as primary outcomes:

- Level of sedation on a sedation assessment scale (as defined/measured by the authors of the trial).
- Numeric rating scale of anxiety or number of participants rated as anxious (as defined/measured by the authors of the trial).
- Proportion of incomplete procedures or where there was difficulty performing the procedures (as defined/measured by the authors of the trial).
- Discomfort/pain (as defined/measured by the authors of the trial).

#### Secondary outcomes

- Anterograde amnesia (defined by number of participants who recalled the procedure).
- Oversedation (as defined/measured by the authors of the trial).
- Disinhibition or excitation (as defined/measured by the authors of the trial).
- Quality of recovery (as defined/measured by the authors of the trial).
- Allergic or anaphylactoid reactions (as defined/measured by the authors of the trial).
- Sedation reversal.
- Tolerance of procedure or participant co-operation (as defined/measured by the authors of the trial).
- Participant or proceduralist satisfaction (as defined/measured by the authors of the trial).

Some of the outcome measures differed between the protocol and the review (see [Differences between protocol and review](#PRO_REV_DIFF)).

## Search methods for identification of studies

### Electronic searches

We searched the Cochrane Central Register of Controlled Trials (CENTRAL) to December 2018, MEDLINE in Ovid (1966 to December 2018) and Ovid Embase (1980 to December 2018).

We combined the sensitive strategies described in Section 6.4 of the *Cochrane Handbook for Systematic Reviews of Interventions* ([Higgins 2011](#REF-Higgins-2011)) to search for RCTs in MEDLINE and Embase.

We searched CENTRAL using the terms given in [Appendix 1](#APP-01). We adapted our MEDLINE search strategy ([Appendix 2](#APP-02)) to reflect the subject headings found in the thesauri used by Embase ([Appendix 3](#APP-03)). We used the free-text terms in all databases and in combination with subject headings when thesauri are a component of a database.

Due to the prolonged editorial process for this update, in May 2020 we conducted a further search of MEDLINE using the terms in [Appendix 2](#APP-02), which were revised from the original to increase sensitivity on recommendation from the Information Specialist. No additional studies were identified for inclusion.

We imposed no language restrictions.

### Searching other resources

For ongoing trials, we searched the following databases on 3 December 2018: metaRegister of Controlled Trials ([www.controlled‐trials.com/mrct](http://www.controlled‐trials.com/mrct)) and Clinical Trials ([clinicaltrials.gov](http://clinicaltrials.gov)). We also screened the reference lists of all eligible trials and reviews.

## Data collection and analysis

### Selection of studies

We screened all titles and abstracts for eligibility. Two authors (AC and JS) independently performed this screening (see [Appendix 4](#APP-04) for a copy of the study selection form). We resolved disagreements by discussion with a third author (KS) to decide on trial inclusion. In the case of insufficient published information to make a decision about inclusion, we attempted to contact the first author of the relevant trial. We compiled a list of eligible trials, each with a unique identifier on a 'Form for eligible trials' (see [Appendix 5](#APP-05)).

### Data extraction and management

Two authors (AC and JS) independently extracted data onto a paper form. A copy of this paper form is in [Appendix 6](#APP-06). We resolved discrepancies by discussion with a third author. AC attempted to contact an author of the relevant trial if we required additional information to include the study. Authors were not contacted to clarify information for risk of bias assessment.

### Assessment of risk of bias in included studies

Two authors (AC and JS) independently assessed the methodological quality of the eligible trials. We resolved disagreements by discussion with a third author.

We performed 'Risk of bias' assessment using the 'Risk of bias' tool as described in Chapter 8 of the *Cochrane Handbook for Systematic Reviews of Intervention*s ([Higgins 2011](#REF-Higgins-2011)) and by [Jüni 2001](#REF-J_x00fc_ni-2001). A copy of the form we used for this is in [Appendix 7](#APP-07).

We assessed each trial according to the quality domains of random sequence generation, allocation concealment, blinding of participants and personnel, blinding of outcome assessment, incomplete outcome data, selective reporting, and any other potential threats to validity.

We considered a trial as having a low risk of bias if we assessed all domains as adequate. We considered a trial as having a high risk of bias if we assessed one or more domains as inadequate or unclear.

We reported the 'Risk of bias' table as part of the [Characteristics of included studies](#CHARACTERISTICS_OF_INCLUDED_STUDIES) table, and present 'Risk of bias' summary figures that detail all of the judgements made for all included studies in the review.

### Measures of treatment effect

For dichotomous variables, we calculated the risk ratio (RR). For continuous variables, we calculated the mean difference (MD) when studies reported their results through the same variables measured with the same instruments (same units of measurement). When continuous data were related to the same clinical effect in the participants but were measured with different instruments (and did not have an interchangeable unit of measurement) we pooled them using the standardized mean difference (SMD). We calculated the 95% confidence interval (CI) as the measure of variance for all statistical methods.

### Unit of analysis issues

To avoid unit of analysis issues, we planned to consider repeated observations as separate outcomes and group them accordingly for analysis ([Morão 2011](#REF-Mor_x00e3_o-2011)). However, the trials included in the review reported the change in time-separated observations (such as the change in pulse oximetry value from before to after the administration of sedation), so we were unable to do this (see [Differences between protocol and review](#PRO_REV_DIFF)). We sought pre-cross-over data for trials that used a cross-over design.

### Dealing with missing data

If trials did not report withdrawals, we assumed there were none. We used an available-case analysis as the default for meta-analysis and we also considered sensitivity analysis using best-case (all participants who withdrew did not experience the event) and worst-case (all participants who withdrew did experience the event) scenarios for any missing data. No outcomes measured with continuous variables had missing data that needed to be included in the meta-analyses.

### Assessment of heterogeneity

We assessed the heterogeneity of included trials as:

1. clinical diversity (e.g. different types of procedures, different forms of midazolam administration, participants' ages, etc.);
2. methodological diversity ('Risk of bias' assessment);
3. statistical heterogeneity (a manifestation of clinical or methodological diversity, or both, among the trials). We assessed statistical heterogeneity with the I² statistic, thereby estimating the percentage of total variance across studies due to heterogeneity rather than chance ([Higgins 2002](#REF-Higgins-2002)). We considered an I² statistic value greater than 50% as considerable heterogeneity or if the Chi² test was significant (see [Data synthesis](#DATA_SYNTHESIS)).

### Assessment of reporting biases

As per the original protocol, we planned to assess publication bias and small-study effects using a funnel plot if there were 10 or more studies included in the meta-analysis ([Morão 2011](#REF-Mor_x00e3_o-2011)). However, we did not perform this analysis because fewer than 10 studies were included in each meta-analysis.

### Data synthesis

We generated meta-analytic estimates for outcomes reported by two or more studies. We performed the analysis using Review Manager 5 ([Review Manager 2014](#REF-Review-Manager-2014)). Because the population is varied, we included all types of procedures. Due to this variation, the intervention effect could have varied across different studies. We, therefore, expected that a random-effects model would be suitable for the meta-analyses. However, a smaller value of the I² statistic (less than 50%) prompted consideration of the use of a fixed-effect model. We performed all analyses according to the intention-to-treat (ITT) principle.

### Subgroup analysis and investigation of heterogeneity

We planned to perform subgroup analyses for age (children, adults (16 years of age or older)), type of procedure (diagnostic, therapeutic) and medical specialty (surgical, non-surgical) ([Morão 2011](#REF-Mor_x00e3_o-2011)). However, there were insufficient studies to conduct subgroup analysis.

### Sensitivity analysis

We planned to perform sensitivity analyses by trials with a low risk of bias versus moderate or high risk of bias ([Morão 2011](#REF-Mor_x00e3_o-2011)). However, we rated most studies to be of either low or very low quality, and this was not appropriate.

### Summary of findings and assessment of the certainty of the evidence

We used the principles of the GRADE system ([Guyatt 2008](#REF-Guyatt-2008)) to assess the quality of the body of evidence associated with the following specific outcomes: level of sedation on a sedation assessment scale (as defined/measured by the authors of the trial); numerical rating of scale of anxiety or number of participants rated as anxious; proportion of incomplete procedures or where there was difficulty performing the procedures; anterograde amnesia (defined by number of participants who recalled the procedure); disinhibition or excitation (as defined/measured by the authors of the trial); discomfort/pain (as defined/measured by the authors of the trial); allergic or anaphylactoid reactions (as defined/measured by the authors of the trial); and we constructed 'Summary of findings' tables using the GRADEpro GDT software ([GRADEpro GDT](#REF-GRADEpro-GDT)).

The GRADE approach appraises the quality of a body of evidence-based on the extent to which one can be confident that an estimate of effect or association reflects the item being assessed. The quality of a body of evidence is based on within-study risk of bias (methodologic quality), the directness of the evidence, heterogeneity of the data, precision of effect estimates and risk of publication bias. The GRADE approach specifies four levels of quality (high, moderate, low, very low). The highest quality rating is for randomized trial evidence, and the lowest is for triple-downgraded randomized trials, downgraded observational studies or case series and case reports.
