# Supplementary material for: Midazolam for sedation before procedures in adults and children: a systematic review update
Source: Syst Rev. 2021 Mar 5;10:69. doi: 10.1186/s13643-021-01617-5 (PMC7936483; doi:10.1186/s13643-021-01617-5)
Supplement: Supplementary file 2 — Additional file 2. Search strategy: Search terms used for the original and updated review [file 13643_2021_1617_MOESM2_ESM.docx]

## 1 CENTRAL search

**Initial search**

#1 MeSH descriptor Midazolam explode all trees
#2 (midazolam near (intranasal or endonasal or intravenous or oral or intramuscular or rectal or sub?lingual)):ti,ab
#3 (#1 OR #2)
#4 MeSH descriptor Conscious Sedation explode all trees
#5 MeSH descriptor Anesthesia Recovery Period explode all trees
#6 MeSH descriptor Anesthesia, Intravenous explode all trees
#7 MeSH descriptor Preanesthetic Medication explode all trees
#8 (anxiolysis or sedat* or pre?medicat* or analges* or surgery or endoscop* or fibroscopy or biopsy or tomography or magnetic resonance or lumbar puncture):ti,ab
#9 (#4 OR #5 OR #6 OR #7 OR #8)
#10 (#3 AND #9)

**Revised search for this update during editorial process (run on May 13th 2020)**

#1    MeSH descriptor: [Midazolam] explode all trees    2992
#2    (midazolam near (intranasal or endonasal or intravenous or oral or intramuscular or rectal or sub?lingual)):ti,ab    2132
#3    MeSH descriptor: [Conscious Sedation] explode all trees    1387
#4    MeSH descriptor: [Anesthesia Recovery Period] explode all trees    2014
#5    MeSH descriptor: [Anesthesia, Intravenous] explode all trees    1904
#6    (anxiolysis or sedat* or analges* or surgery or endoscop* or fibroscopy or biopsy or tomography or magnetic resonance or lumbar puncture):ti,ab    245315
#7    #3 or #4 or #5 or #6    246496
#8    #1 or #2    4161
#9    #7 and #8    3157
#10    #9 with Cochrane Library publication date Between Oct 2018 and May 2020, in Trials    465

## 2 Ovid MEDLINE search

**Initial search**

1. Midazolam/ or (midazolam adj5 (intranasal or endonasal or intravenous or oral or intramuscular or rectal or sub?lingual)).ti,ab.
2. Conscious Sedation/ or Anesthesia Recovery Period/ or Anesthesia, Intravenous/ or exp Preanesthetic Medication/ or (anxiolysis or sedat* or pre?medicat* or analges* or surgery or endoscop* or fibroscopy or biopsy or tomography or magnetic resonance or lumbar puncture).ti,ab.
3. 1 and 2
4. ((randomized controlled trial or controlled clinical trial).pt. or randomized.ab. or placebo.ab. or clinical trials as topic.sh. or randomly.ab. or trial.ti.) not (animals not (humans and animals)).sh.
5. 3 and 4

**Revised search for this update during editorial process (run on May 13th 2020)**

1.     Midazolam/ or (midazolam adj5 (intranasal or endonasal or intravenous or oral or intramuscular or rectal or sub?lingual)).ti,ab.

2.     Conscious Sedation/ or Anesthesia Recovery Period/ or Anesthesia, Intravenous/ or (anxiolysis or sedat* or pre?medicat* or analges* or surgery or endoscop* or fibroscopy or biopsy or tomography or magnetic resonance or lumbar puncture).ti,ab.

3.     ((randomized controlled trial or controlled clinical trial).pt. or randomized.ab. or placebo.ab. or clinical trials as topic.sh. or randomly.ab. or trial.ti.) not (animals not (humans and animals)).sh.

4.     1 AND 2 AND 3

5. (2018* or 2019* or 2020*).dt,ez,yr,dp,ed.

6. 4 AND 5

## 3 Ovid Embase search

**Initial search**

1. midazolam.ti,ab.
2. sedation/ or anesthetic recovery/ or intravenous anesthesia/ or premedication/ or (anxiolysis or sedat* or pre?medicat* or analges* or surgery or endoscop* or fibroscopy or biopsy or tomography or magnetic resonance or lumbar puncture).ti,ab.
3. 1 and 2
4. (placebo.sh. or controlled study.ab. or random*.ti,ab. or trial*.ti,ab.) not (animals not (humans and animals)).sh.
5. 3 and 4

**Revised search for this update during editorial process (run on May 13th 2020)**

1.     midazolam.ti,ab. 
2.     sedation/ or anesthetic recovery/ or intravenous anesthesia/ or (anxiolysis or sedat* or analges* or surgery or endoscop* or fibroscopy or biopsy or tomography or magnetic resonance or lumbar puncture).ti,ab. 
3.     1 and 2 
4     (placebo.sh. or controlled study.ab. or random*.ti,ab. or trial*.ti,ab.) not (animals not (humans and animals)).sh.
5.    3 and 4

6.  (2018* or 2019* or 2020*).dc,dp,yr.

7. 5 and 6
