# Supplementary material for: Midazolam for sedation before procedures in adults and children: a systematic review update
Source: Syst Rev. 2021 Mar 5;10:69. doi: 10.1186/s13643-021-01617-5 (PMC7936483; doi:10.1186/s13643-021-01617-5)
Supplement: Supplementary file 3 — Additional file 3. Study characteristics: Sample and intervention characteristics and outcome descriptions as well as risk of bias assessments for each study included in the updated review [file 13643_2021_1617_MOESM3_ESM.docx]

# Characteristics of studies

## Characteristics of included studies

### Akil 2005

| **Methods** | Parallel-group single-centre randomized controlled trial conducted in Turkey between September 2002 to April 2003 |
| --- | --- |
| **Participants** | 53 children requiring micturating cystourethrogram with sedation (39 girls, 14 boys; mean age of 5.8 ± 3.5 years) |
| **Interventions** | 1. Oral midazolam 0.6 mg/kg (max 15 mg) versus chloral hydrate 25 mg/kg (max 0.5 g) and placebo (saline) 15 - 30 minutes before procedure |
| **Outcomes** | **Measured during procedures**   1. Incomplete procedures 2. Anxiety (measured during the procedure using Spielberger's Trait Anxiety Inventory) 3. Participant co-operation (measured during the procedure using Houpt behavioural scale; range 1 - 6 with higher scores indicating better co-operation) 4. Tolerance of procedure (measured during the procedure using Frankl behaviour rating score; range 1 - 4 with higher scores indicating better tolerance |
| **Identification** |  |
| **Notes** | Conflicts of interest or funding sources were not reported |

#### Risk of bias table

| **Bias** | **Authors' judgement** | **Support for judgement** |
| --- | --- | --- |
| Random sequence generation (selection bias) | Unclear risk | Not reported (not contacted for clarification) |
| Allocation concealment (selection bias) | Unclear risk | Not reported (not contacted for clarification) |
| Blinding of participants and personnel (performance bias) | High risk | Not blinded |
| Blinding of outcome assessment (detection bias) | High risk | Outcomes included in this review were not blinded - only assessment of image quality was performed by a blinded outcome assessor |
| Incomplete outcome data (attrition bias) | Low risk | No withdrawals |
| Selective reporting (reporting bias) | Unclear risk | No clear evidence that measured outcomes were not reported (trial protocols were not sought for confirmation) |
| Other bias | Low risk | None expected |

### Aktogu 1994

| **Methods** | Parallel-group single-centre randomized controlled trial conducted in Turkey |
| --- | --- |
| **Participants** | 32 adults undergoing bronchoscopy (mean age midazolam group 49.4 ± 13.3 and diazepam group 50.9 ± 12.1; 50% men in both groups) |
| **Interventions** | 1. Midazolam 0.06 mg/kg administered intravenously 2. Diazepam 0.15 mg/kg administered intravenously |
| **Outcomes** | **Measured 5 and 10 minutes after start of procedure**   1. Level of sedation (only the percentage of participants who scored in the 'awake' rank of a sedation scale that ranged from 0 to 4 (0 = awake and 4 = reactive to pain but no verbal communication) that was measured 5 minutes after sedation was administered)   **Measured 24 hours after procedure**   1. Anterograde amnesia (defined by number of participants who recalled the procedure) |
| **Identification** |  |
| **Notes** | Conflicts of interest or funding sources were not reported. |

#### Risk of bias table

| **Bias** | **Authors' judgement** | **Support for judgement** |
| --- | --- | --- |
| Random sequence generation (selection bias) | Unclear risk | Unclear because there was no mention of randomization method, just a statement that participants were randomized (not contacted for clarification) |
| Allocation concealment (selection bias) | Unclear risk | Unclear because there was no mention of allocation method, just a statement that participants were randomized (not contacted for clarification) |
| Blinding of participants and personnel (performance bias) | Unclear risk | Unclear (not contacted for clarification) |
| Blinding of outcome assessment (detection bias) | Unclear risk | Only statistical analysis of the questionnaires was reported to have been performed by a blinded statistician |
| Incomplete outcome data (attrition bias) | Low risk | No withdrawals |
| Selective reporting (reporting bias) | Unclear risk | No clear evidence that measured outcomes were not reported (trial protocols were not sought for confirmation) |
| Other bias | Low risk | None expected |

### Alp 2019

| **Methods** | **Study design:** Randomized controlled trial  **Study grouping:** Parallel group |
| --- | --- |
| **Participants** | **Baseline Characteristics**   - *Males (%)*: 102 (47%) - *Median age*: 22 (range 9-38)   **Included criteria:** Patients within 9–36 months of age who were referred to paediatric cardiology for further evaluation of a heart murmur were enrolled. The inclusion criteria were full consciousness before transthoracic echocardiography, lack of hypotonia, and a successfully completed procedure.  **Excluded criteria:** Exclusion criteria were history of associated drug hypersensitivity, presence of neurologic deficits and/or developmental delay, presence of respiratory distress, presence of upper or lower respiratory system infection, gastroenteritis with/ without vomiting, presence of genetic diseases such as Down syndrome, presence of any disease that suggests pulmonary hypertension or history of pulmonary hypertension, current medications and/or associated medical conditions known to contraindicate sedation, instability of patient’s vital signs, meanwhile intensive care unit stay, detection of pulmonary hypertension, and failure to complete the procedure. |
| **Interventions** | **Intervention Characteristics**  Intranasal midazolam 0.2mg/kg (max 3mg)  Intranasal Ketamine 4mg/kg (max 100mg) |
| **Outcomes** | *Incomplete procedure*   - **Outcome type**: DichotomousOutcome   *Sedation*   - **Outcome type**: DichotomousOutcome - **Reporting**: Fully reported - **Scale**: RASS - **Range**: 'Alert and calm', 'Drowsy', 'Sedation'. - **Unit of measure**: Defined as number of participants rated as 'sedated' - **Direction**: Higher is better - **Data value**: Endpoint |
| **Identification** | **Sponsorship source:** This research received no specific grant from any funding agency, commercial, or not-for-profit sectors.  **Country:** Turkey  **Setting:** Paediatric echocardiography  **Comments:**  **Authors name:** Hayrullah Alp, MD  **Institution:** Dr Ali Kemal Belviranlı Obstetrics and Children’s Hospital  **Email:** drhayrullahalp@hotmail.com  **Address:** Fatih Mah. Yeni İstanbul Cd. No: 32, Selçuklu,Konya 42285, Turkey |
| **Notes** |  |

#### Risk of bias table

| **Bias** | **Authors' judgement** | **Support for judgement** |
| --- | --- | --- |
| Random sequence generation (selection bias) | High risk | Judgement Comment: No detail provided about how the sequence was generated. |
| Allocation concealment (selection bias) | High risk | Judgement Comment: No detail provided about how the allocation was concealed. |
| Blinding of participants and personnel (performance bias) | High risk | Judgement Comment: Not blinded |
| Blinding of outcome assessment (detection bias) | Low risk | Quote: "Children were assessed as per the scale on 15th, 30th, 45th, and 60th minutes by two paediatricians who were blinded to the study." |
| Incomplete outcome data (attrition bias) | Low risk | Judgement Comment: Results were fully reported |
| Selective reporting (reporting bias) | Unclear risk | Judgement Comment: Trial not registered |
| Other bias | Low risk | Judgement Comment: None |

### Bell 1988

| **Methods** | Parallel-group single-centre randomized controlled trial conducted in the UK |
| --- | --- |
| **Participants** | 102 adults undergoing upper gastrointestinal endoscopy (mean age midazolam group was 62.8 ± 16.1 years and 65.8 ± 11.5 in the diazepam group) |
| **Interventions** | 1. Intravenous midazolam 2.5 mg or 1 mg for elderly (mean 6.0 ± 2.8) vs intravenous diazepam 5 mg or 2 mg for elderly (mean 11.5 ± 6.7) |
| **Outcomes** |  |
| **Identification** |  |
| **Notes** | Conflicts of interest or funding sources were not reported |

#### Risk of bias table

| **Bias** | **Authors' judgement** | **Support for judgement** |
| --- | --- | --- |
| Random sequence generation (selection bias) | Unclear risk | Not reported (not contacted for clarification) |
| Allocation concealment (selection bias) | Unclear risk | No information (not contacted for clarification) |
| Blinding of participants and personnel (performance bias) | Unclear risk | Unclear how blinding was performed in the study (The patients were allocated to be sedated either with  intravenous diazepam (Diazemuls- KabiVitrum) or intravenous midazolam( Hypnovel, Roche) by the nursing staff who drew up one or other sedative independently of the endoscopist.) |
| Blinding of outcome assessment (detection bias) | Unclear risk | Unclear how blinding was performed in the study |
| Incomplete outcome data (attrition bias) | Low risk | No withdrawals |
| Selective reporting (reporting bias) | Unclear risk | No clear evidence that measured outcomes were not reported (trial protocols were not sought for confirmation) |
| Other bias | Low risk | None expected |

### Bhalla 2006

| **Methods** | Parallel-group single-centre randomized controlled trial conducted in India from January to July 2000. |
| --- | --- |
| **Participants** | 252 adults undergoing diagnostic or therapeutic upper gastrointestinal endoscopy. Authors stated that there were no difference in baseline characteristics of the 3 groups |
| **Interventions** | 1. Intravenous midazolam 5 mg (3 mg if older than 65) 2. Intravenous diazepam 5 mg (3 mg if older than 65) 3. Intravenous saline (placebo) |
| **Outcomes** | **Measured during the procedure**   1. Incomplete procedures 2. Difficulty performing procedure 3. Discomfort/pain (as defined/measured by the authors of the trial) |
| **Identification** |  |
| **Notes** | Conflicts of interest or funding sources were not reported |

#### Risk of bias table

| **Bias** | **Authors' judgement** | **Support for judgement** |
| --- | --- | --- |
| Random sequence generation (selection bias) | Low risk | Quote: "Block randomisation" |
| Allocation concealment (selection bias) | Unclear risk | Not reported (not contacted for clarification) |
| Blinding of participants and personnel (performance bias) | Low risk | Endoscopist and investigator recording haemodynamic data were not aware of the nature of the medications |
| Blinding of outcome assessment (detection bias) | Low risk | Endoscopist and investigator recording haemodynamic data were not aware of the nature of the medications |
| Incomplete outcome data (attrition bias) | Low risk | No loss to follow-up reported |
| Selective reporting (reporting bias) | Unclear risk | No clear evidence that measured outcomes were not reported (trial protocols were not sought for confirmation) |
| Other bias | Low risk | None expected |

### Bianchi Porro 1988

| **Methods** | Single-centre cross-over randomized controlled trial conducted in Italy |
| --- | --- |
| **Participants** | 23 adults undergoing upper gastrointestinal endoscopy (14 men and 9 women; mean weight 60.7 kilograms; age range 20 to 48 years; mean age 32.5) |
| **Interventions** | 1. Intravenous midazolam 0.07 mg/kg 2. Intravenous diazepam 0.15 mg/kg |
| **Outcomes** | **Measured during the procedure**   1. Level of sedation using a sedation assessment scale 2. Participant co-operation   **Measured 2 hours after the procedure**   1. Quality of recovery   **Measured 24 hours after the procedure**   1. Anterograde amnesia (defined by number of participants who recalled the procedure) |
| **Identification** |  |
| **Notes** | At least 30 days between procedures  We were unable to locate contact details of the authors to access pre-cross-over data  Conflicts of interest or funding sources were not reported |

#### Risk of bias table

| **Bias** | **Authors' judgement** | **Support for judgement** |
| --- | --- | --- |
| Random sequence generation (selection bias) | Unclear risk | Not reported (we were unable to locate the contact details of the authors to request further information) |
| Allocation concealment (selection bias) | Unclear risk | Not reported (we were unable to locate the contact details of the authors to request further information) |
| Blinding of participants and personnel (performance bias) | Low risk | Drugs prepared and administered by physician not performing endoscopy or assessments |
| Blinding of outcome assessment (detection bias) | Low risk | Drugs prepared and administered by physician not performing endoscopy or assessments |
| Incomplete outcome data (attrition bias) | Low risk | No withdrawals |
| Selective reporting (reporting bias) | Unclear risk | No clear evidence that measured outcomes were not reported (trial protocols were not sought for confirmation) |
| Other bias | Low risk | None expected |

### Cole 1983

| **Methods** | Parallel-group randomized controlled trial conducted in 2 sites in the USA |
| --- | --- |
| **Participants** | 40 adult participants (American Society of Anesthesiology physical classification status I - II) undergoing upper gastrointestinal endoscopy for the first time (aged 18 - 70 years; authors reported that the groups were comparable in all parameters evaluated: age, sex, weight, race (white/non-white), psychoactive drug history, duration of procedure, injection speed, data collection site) |
| **Interventions** | 1. Intravenous midazolam 5 mg bolus with 2.5 - 3.75 mg increments at 30 -60-second intervals as required. Half doses for elderly 2. Same dose for diazepam |
| **Outcomes** | **Measured during the procedure**   1. Discomfort/pain (as defined/measured by the authors of the trial) (measured in the study as absolute number with pain)   **Measured after the procedure**   1. Proceduralist satisfaction   **Measured the day after the procedure**   1. Participant satisfaction 2. Quality of recovery (number of participants reporting unusual sensations the day after the procedure) 3. Anterograde amnesia (numerical rating from 0 to 100 with a lower number indicating greater amnesia) |
| **Identification** |  |
| **Notes** | Authors declared that Hoffman-La Riche Inc. provided financial assistance for the study, but did not disclose the role of the funder in the study |

#### Risk of bias table

| **Bias** | **Authors' judgement** | **Support for judgement** |
| --- | --- | --- |
| Random sequence generation (selection bias) | Unclear risk | Not reported (not contacted for clarification) |
| Allocation concealment (selection bias) | Unclear risk | Not reported (not contacted for clarification) |
| Blinding of participants and personnel (performance bias) | Low risk | Both the participants and the endoscopist were masked to treatment allocation |
| Blinding of outcome assessment (detection bias) | Low risk | Both the participant and the endoscopist were masked to treatment allocation |
| Incomplete outcome data (attrition bias) | Low risk | No withdrawals |
| Selective reporting (reporting bias) | Unclear risk | No clear evidence that measured outcomes were not reported (trial protocols were not sought for confirmation) |
| Other bias | Low risk | None expected |

### Coll-Vinent 2003

| **Methods** | Single-centre parallel-group randomized controlled trial conducted in Spain |
| --- | --- |
| **Participants** | 32 consecutive adults undergoing cardioversion for supraventricular arrhythmia in an emergency department |
| **Interventions** | **Randomized to 1 of 4 treatment arms (all intravenous administration)**   1. 0.2 mg/kg midazolam 2. 0.2 mg/kg etomidate 3. 1.5 mg/kg propofol 4. 0.2 mg/kg midazolam and flumazenil 0.5 mg bolus followed by 0.5 mg infusion for 1 hour   If induction was not achieved within 3 - 5 minutes, supplementary doses of etomidate (0.05 mg/kg), midazolam (0.05 mg/kg) or propofol (0.5 mg/kg) were injected at 1-minute intervals until the desired effect was obtained |
| **Outcomes** | **Measured during the procedure**   1. Level of sedation using a sedation assessment scale (Ramsay scale) |
| **Identification** |  |
| **Notes** | Only median values reported for continuous outcomes (skewed distributions because of small sample size). We did not include the midazolam and flumazenil group in our review because combinations of medications with midazolam were excluded. The authors declared that no outside funding or support was received for the study. No other conflicts of interest were reported. |

#### Risk of bias table

| **Bias** | **Authors' judgement** | **Support for judgement** |
| --- | --- | --- |
| Random sequence generation (selection bias) | Low risk | Random-number table |
| Allocation concealment (selection bias) | Low risk | Sealed envelopes |
| Blinding of participants and personnel (performance bias) | High risk | Not blinded |
| Blinding of outcome assessment (detection bias) | High risk | Not blinded |
| Incomplete outcome data (attrition bias) | Low risk | No withdrawals |
| Selective reporting (reporting bias) | Unclear risk | No clear evidence that measured outcomes were not reported (trial protocols were not sought for confirmation) |
| Other bias | Low risk | None expected |

### Córdova 1992

| **Methods** | Single-centre parallel-group randomized controlled trial conducted in Mexico |
| --- | --- |
| **Participants** | 60 adults aged 18 - 65 years who underwent upper digestive endoscopy. Participants with allergy to midazolam or diazepam were excluded |
| **Interventions** | 1. Intravenous diazepam (0.15 mg/kg) 2. Intravenous midazolam (0.10 mg/kg) |
| **Outcomes** | **Measured during the procedure**   1. Tolerance of procedure   **Measured after the procedure**   1. Anterograde amnesia (defined by number of participants who recalled the procedure) |
| **Identification** |  |
| **Notes** | Conflicts of interest or funding sources were not reported |

#### Risk of bias table

| **Bias** | **Authors' judgement** | **Support for judgement** |
| --- | --- | --- |
| Random sequence generation (selection bias) | Unclear risk | No explanation about method to generate allocation. Just stated (quote:) “randomly allocated to two groups” (not contacted for clarification) |
| Allocation concealment (selection bias) | Unclear risk | Authors describe no method for allocation concealment (not contacted for clarification) |
| Blinding of participants and personnel (performance bias) | Unclear risk | Methods include a mention of “double blinding”, but no further details about who and how |
| Blinding of outcome assessment (detection bias) | Unclear risk | Method include a mention of “double blinding”, but no further details about who and how |
| Incomplete outcome data (attrition bias) | Low risk | No withdrawals |
| Selective reporting (reporting bias) | Unclear risk | No clear evidence that measured outcomes were not reported (trial protocols were not sought for confirmation) |
| Other bias | Low risk | None expected |

### D'Agostino 2000

| **Methods** | Single-centre parallel-group randomized controlled trial conducted in USA from June 1994 to August 1995 (14 months). |
| --- | --- |
| **Participants** | 40 2-months – 8-year-old children requiring neuroimaging with sedation. Average age 31 ± 23 months and 45% were boys. |
| **Interventions** | 1. Oral midazolam 0.5 mg/kg (max 10 mg) vs oral chloral hydrate 75 mg/kg (max 2 g)   Could received additional dose (50% of original dose) if required |
| **Outcomes** | **Measured during the procedure**   1. Numerical rating of anxiet 2. Incomplete procedures |
| **Identification** |  |
| **Notes** | Conflicts of interest or funding sources were not reported |

#### Risk of bias table

| **Bias** | **Authors' judgement** | **Support for judgement** |
| --- | --- | --- |
| Random sequence generation (selection bias) | Low risk | Random-number table |
| Allocation concealment (selection bias) | Unclear risk | Not reported (not contacted for clarification) |
| Blinding of participants and personnel (performance bias) | Low risk | Children were administered freshly-prepared, identically-appearing, cherry-flavoured liquids in body weight equivalent volumes |
| Blinding of outcome assessment (detection bias) | Low risk | Neither the participant nor any of the investigators were aware of the active component given to individual participants |
| Incomplete outcome data (attrition bias) | Low risk | Reasons for withdrawals were reported. Randomized children who did not complete the protocol included 1 with respiratory distress, 1 who ate a full meal prior to intended drug administration, 1 who fell asleep after intravenous line placement and 4 who cancelled their appointments after randomization |
| Selective reporting (reporting bias) | Unclear risk | No clear evidence that measured outcomes were not reported (trial protocols were not sought for confirmation) |
| Other bias | Low risk | None expected |

### De Alencar 2010

| **Methods** | Single-centre parallel-group randomized controlled trial conducted in Brazil |
| --- | --- |
| **Participants** | 70 adult participants (American Society of Anesthesiology physical classification status I - II) undergoing lower eyelid blepharoplasty under local anaesthetic. Mean age in the groups ranged from 57.9 ± 10.2 years to 64 ± 14.4 years. There was more women than men in each group. |
| **Interventions** | **Intravenous administration of**   1. diazepam 10 mg and clonidine 0.15 mg 2. diazepam 10 mg 3. Midazolam 15 mg 4. Midazolam 15 mg and clonidine 0.15 mg (not used in this review) |
| **Outcomes** | **Measured before the procedure**   1. Level of sedation using a sedation assessment scale (Michigan University Scale range from 0 = awake to 4 = unarousable to stimuli) |
| **Identification** |  |
| **Notes** | Conflicts of interest or funding sources were not reported |

#### Risk of bias table

| **Bias** | **Authors' judgement** | **Support for judgement** |
| --- | --- | --- |
| Random sequence generation (selection bias) | Unclear risk | Not reported (not contacted for clarification) |
| Allocation concealment (selection bias) | Unclear risk | Not reported (not contacted for clarification) |
| Blinding of participants and personnel (performance bias) | High risk | Not blinded |
| Blinding of outcome assessment (detection bias) | High risk | Not blinded |
| Incomplete outcome data (attrition bias) | Low risk | No withdrawals |
| Selective reporting (reporting bias) | Unclear risk | No clear evidence that measured outcomes were not reported (trial protocols were not sought for confirmation) |
| Other bias | Low risk | None expected |

### Demiraran 2007a

| **Methods** | **Study design:** Randomized controlled trial  **Study grouping:** Parallel group |
| --- | --- |
| **Participants** | **Baseline Characteristics**  Intravenous midazolam   - *Males (%)*: 36 - *mean age*: 43.3   Dexmedetomidine   - *Males (%)*: 52 - *mean age*: 42.2   Overall   - *Males (%)*: 44 - *mean age*: 42.75   **Included criteria:** Age 18-60 years, who rated I and II on the American Society of Anesthesiologists physical status classification system.  **Excluded criteria:** Patients younger than 18 years of age. Presence of hypertension, prior gastrectomy, psychiatric disease or long-term psychiatric drug addiction, chronic use or addiction to opiates or sedatives, and neoplastic or other serious concomitant diseases. Previous adverse reactions to any medication used in the study, a baseline SBP less than 90 mmHg. An American Society of Anesthesiologists physical status classification of III, IV, or V, pain in the GI tract and those who were pregnant.  **Pretreatment:** |
| **Interventions** | **Intervention Characteristics**  Intravenous midazolam   - *Dose*: 0.07 mg/kg (total dose 5 mg)   Dexmedetomidine   - *Dose*: 1 µg/kg IV over 10 min before procedure, followed by an infusion of dexmedetomidine 0.2 µg/kg/h IV. |
| **Outcomes** | *Discomfort (Patient-rated visual analogue scale scores)*   - **Outcome type**: ContinuousOutcome - **Scale**: Visual Analogue Scale - **Range**: 0=none, 100=severe - **Direction**: Lower is better - **Data value**: Endpoint   *Satisfaction (Patient-rated visual analogue scale scores)*   - **Outcome type**: ContinuousOutcome - **Scale**: Visual Analogue Scale - **Range**: 0=very dissatisfied, 100=extremely satisfied - **Direction**: Higher is better   *Anxiety (Patient-rated visual analogue scale scores)*   - **Outcome type**: ContinuousOutcome - **Scale**: Visual Analogue Scale - **Range**: 0=none, 100=severe - **Direction**: Lower is better   *Discomfort (Endoscopist visual analogue scale score)*   - **Outcome type**: ContinuousOutcome - **Scale**: Visual Analogue Scale - **Range**: 0=none, 100=severe - **Direction**: Lower is better   *Overall satisfaction with patient's sedation level (Endoscopist visual analogue scale scores)*   - **Outcome type**: ContinuousOutcome - **Scale**: Visual Analogue Scale - **Range**: 0=very dissatisfied, 100=very satisfied - **Direction**: Higher is better   *Technical difficulty (Endoscopist visual analogue scale scores)*   - **Outcome type**: ContinuousOutcome - **Scale**: Visual Analogue Scale - **Range**: 0=easy, 100=very demanding - **Direction**: Lower is better |
| **Identification** | **Sponsorship source:** No funding reported  **Country:** Turkey  **Setting:** Endoscopy  **Comments:**  **Authors name:** Yavuz Demiraran  **Institution:** Abant Izzet Baysal University  **Email:** demiryvz@yahoo.com  **Address:** Kat: 3, Daire: 24, Akcakoca, Duzce, Turkey 81100 |
| **Notes** |  |

#### Risk of bias table

| **Bias** | **Authors' judgement** | **Support for judgement** |
| --- | --- | --- |
| Random sequence generation (selection bias) | Low risk | Quote: "A computer-generated randomization list was used to assign patients to one of two groups." |
| Allocation concealment (selection bias) | High risk | Judgement Comment: No information provided about allocation concealment |
| Blinding of participants and personnel (performance bias) | High risk | Quote: "The randomization was conducted in the procedure room; thus, the endoscopist and the procedure room personnel were not blinded to the patient’s sedation regimen." |
| Blinding of outcome assessment (detection bias) | Low risk | Quote: "The same research nurse, who was blinded to the type of sedation, conducted all evaluations before and after the procedures." |
| Incomplete outcome data (attrition bias) | Low risk | Quote: "All 50 patients (25 in each group) completed the study." |
| Selective reporting (reporting bias) | Low risk | Judgement Comment: Results for outcomes were reported |
| Other bias | Low risk | Judgement Comment: None identified |

### Derakhshanfar 2013

| **Methods** | Single-centre parallel-group randomized controlled trial conducted in Iran from June to November 2010. |
| --- | --- |
| **Participants** | 160 children aged 2 to 7 years requiring lumbar puncture (mean age: 3.4 ± 1.9 years old in chloral hydrate, 3.6 ± 2.6 years old in midazolam group; 42 girls and 38 boys in chloral hydrate group and 48 girls and 32 boys in the midazolam group) |
| **Interventions** | **Oral administration of**   1. 80 mg/kg chloral hydrate followed by further dose 20 mg/kg if required 20 minutes later 2. 0.5 mg/kg midazolam. Additional dose up to 8 mg for inadequate sedation |
| **Outcomes** | **Measured before the procedure**   1. Level of sedation using a sedation assessment scale (Wheeler's sedation level with scores ranging from 1 = agitated to 4 = eyes closing spontaneously but response to minor stimuli 2. Incomplete procedures 3. Disinhibition/excitation   **Measured after the procedure**   1. Quality of recovery (prolonged sedation) |
| **Identification** |  |
| **Notes** | Conflicts of interest or funding sources were not reported |

#### Risk of bias table

| **Bias** | **Authors' judgement** | **Support for judgement** |
| --- | --- | --- |
| Random sequence generation (selection bias) | Unclear risk | Not reported (not contacted for clarification) |
| Allocation concealment (selection bias) | Unclear risk | Not reported (not contacted for clarification) |
| Blinding of participants and personnel (performance bias) | High risk | Although the investigator, proceduralist and nurse were "unaware" of the drug used, the parents (who also rated sedation) did know which |
| Blinding of outcome assessment (detection bias) | High risk | Although the investigator, proceduralist and nurse were "unaware" of the drug used, the parents (who also rated sedation) did know which medication was administered. |
| Incomplete outcome data (attrition bias) | Low risk | No withdrawals |
| Selective reporting (reporting bias) | Unclear risk | No clear evidence that measured outcomes were not reported (trial protocols were not sought for confirmation) |
| Other bias | Low risk | None expected |

### Eisapour 2015

| **Methods** | **Study design:** Randomized controlled trial  **Study grouping:** Parallel group |
| --- | --- |
| **Participants** | **Baseline Characteristics**  Intravenouos midazolam   - *Males (%)*: 10 (62.5) - *Mean age (months)*: 19 (9)   Placebo   - *Males (%)*: 7 (43.8) - *Mean age (months)*: 23.3 (14.9)   Overall   - *Males (%)*: - *Mean age (months)*:   **Included criteria:** 6-months to 4-year children referred to Amirkola Hospital who had a defi-nite diagnosis of ileocolic intussusception based on so-nographic findings  **Excluded criteria:** Exclusion criteria included the presence of an underlying pathologic factor (Lead point) for Intussusception, passage of more than 48 hours of the onset of symptoms, the presence of signs of peritonitis or bowel perforation, dehydration, lethargy, history of a known gastrointestinal disease such as celiac disease or cystic fibrosis, history of a chronic hypoxic pulmonary disease, cyanotic heart disease, history of previous intussusception and the history of allergy to benzodiazepines.  **Pretreatment:** |
| **Interventions** | **Intervention Characteristics**  Intravenouos midazolam 0.1mg/kg  Placebo |
| **Outcomes** | *Incomplete procedure*   - **Outcome type**: DichotomousOutcome |
| **Identification** | **Sponsorship source:** No conflicts reported  **Country:** Iran  **Setting:** Amirkola Children's Hospital  **Comments:**  **Authors name:** Ali Eisapour  **Institution:** Non-Communicable pediatric diseases research center, Babol University of medical science  **Email:** r.mehrayin@hotmail.com  **Address:** Shafizade Children's Hospital, Amirkola, Babol4731741151 |
| **Notes** |  |

#### Risk of bias table

| **Bias** | **Authors' judgement** | **Support for judgement** |
| --- | --- | --- |
| Random sequence generation (selection bias) | High risk | Quote: "32 eligible patients were ran- domly assigned (every second one) to the study and con- trol groups." |
| Allocation concealment (selection bias) | Unclear risk | Judgement Comment: Just stated that it was a 'double-blind clinical trial' |
| Blinding of participants and personnel (performance bias) | Unclear risk | Judgement Comment: Just stated that it was a 'double-blind clinical trial' |
| Blinding of outcome assessment (detection bias) | Unclear risk | Judgement Comment: Just stated that it was a 'double-blind clinical trial' |
| Incomplete outcome data (attrition bias) | Low risk | Judgement Comment: Outcomes were fully reported |
| Selective reporting (reporting bias) | Low risk | Quote: "(Clinical Trial Registry Number: IRCT138904264395N1),"  Judgement Comment: Outcomes match registry |
| Other bias | Low risk | Judgement Comment: None expected |

### Everitt 2002

| **Methods** | Single-centre parallel-group randomized controlled trial conducted in Australia from April to December 1997. |
| --- | --- |
| **Participants** | 129 children aged 1 - 4 years with uncomplicated lacerations that required 2 or more sutures (42 excluded from the review due to comparison between different routes of administration). Similar at baseline for age, heart rate, respiratory rate, blood pressure, oxygen saturation, anxiety score and laceration characteristics (summary statistics were not reported) |
| **Interventions** | **Oral administration of**   1. 0.5 mg/kg diazepam 2. 1 mg/kg midazolam 3. 0.4 mg/kg intranasal midazolam (not included) |
| **Outcomes** | **Measured during the procedure immediately after suturing**   1. Level of sedation rated on a scale of 0 to 100 (lower score = better sedation) by proceduralist, nurse, parent and investigator   **Measured 24 to 48 hours after the procedure**   1. Quality of recovery (proportion of participants who were 'drowsy' at home) |
| **Identification** |  |
| **Notes** | Conflicts of interest or funding sources were not reported |

#### Risk of bias table

| **Bias** | **Authors' judgement** | **Support for judgement** |
| --- | --- | --- |
| Random sequence generation (selection bias) | Unclear risk | Not reported (not contacted for clarification) |
| Allocation concealment (selection bias) | Unclear risk | Not reported (not contacted for clarification) |
| Blinding of participants and personnel (performance bias) | Low risk | A nurse not involved in the participants' care performed the drug administration. The investigator (I.J.E.), suturing doctor, and nurse assisting with suturing were unaware which sedative had been given. Parents were asked not to reveal which drug or route of delivery their child had received |
| Blinding of outcome assessment (detection bias) | Low risk | A nurse not involved in the participants' care performed the drug administration. The investigator (I.J.E.), suturing doctor, and nurse assisting with suturing were unaware which sedative had been given. Parents were asked not to reveal which drug or route of delivery their child had received |
| Incomplete outcome data (attrition bias) | Unclear risk | Unclear how much data were missing for parents' assessments |
| Selective reporting (reporting bias) | Unclear risk | No clear evidence that measured outcomes were not reported (trial protocols were not sought for confirmation) |
| Other bias | Low risk | None expected |

### Fakheri 2010

| **Methods** | Single-centre parallel-group randomized controlled trial conducted in Iran from April to July 2008. |
| --- | --- |
| **Participants** | 180 adults over 18 years of age without serious comorbidities undergoing upper gastrointestinal endoscopy (mean age: 46.9 ± 17.5 years in midazolam group and 47 ± 17.5 years in placebo group; 49% men in midazolam group and 43% men in placebo group) |
| **Interventions** | 1. Intravenous midazolam - mean dose 3.2 (1.6) mg 2. Saline placebo |
| **Outcomes** | **None** |
| **Identification** |  |
| **Notes** | Conflicts of interest or funding sources were not reported |

#### Risk of bias table

| **Bias** | **Authors' judgement** | **Support for judgement** |
| --- | --- | --- |
| Random sequence generation (selection bias) | Unclear risk | Not reported (not contacted for clarification) |
| Allocation concealment (selection bias) | Unclear risk | Not reported (not contacted for clarification) |
| Blinding of participants and personnel (performance bias) | High risk | Not blinded |
| Blinding of outcome assessment (detection bias) | High risk | Not blinded |
| Incomplete outcome data (attrition bias) | High risk | Excluded participants who required more than 10 mg midazolam for sedation |
| Selective reporting (reporting bias) | Unclear risk | No clear evidence that measured outcomes were not reported (trial protocols were not sought for confirmation) |
| Other bias | Unclear risk | None expected |

### Gilvarry 1990

| **Methods** | Single-centre parallel-group randomized controlled trial conducted in the UK |
| --- | --- |
| **Participants** | 60 adults undergoing upper gastrointestinal endoscopy (mean age: 41.4 in diazepam group and 42.2 in midazolam group; 46% men in diazepam group and 43% men in diazepam group) |
| **Interventions** | 1. 10 mg intravenous midazolam 2. 20 mg intravenous diazepam |
| **Outcomes** | **Measured 24 hours after the procedure**   1. Participant satisfaction (measured in the trial as the participant considered sedation was inadequate) 2. Anterograde amnesia (recalled procedures) |
| **Identification** |  |
| **Notes** | Conflicts of interest or funding sources were not reported |

#### Risk of bias table

| **Bias** | **Authors' judgement** | **Support for judgement** |
| --- | --- | --- |
| Random sequence generation (selection bias) | Unclear risk | Quote: "Stratified randomised order" |
| Allocation concealment (selection bias) | Unclear risk | Not reported (not contacted for clarification) |
| Blinding of participants and personnel (performance bias) | High risk | Not blinded |
| Blinding of outcome assessment (detection bias) | Low risk | An author who did not know which medication had been administered assessed outcomes 24 hours after the procedure |
| Incomplete outcome data (attrition bias) | Low risk | No withdrawals |
| Selective reporting (reporting bias) | Unclear risk | No clear evidence that measured outcomes were not reported (trial protocols were not sought for confirmation) |
| Other bias | Low risk | None expected |

### Hollenhorst 2001

| **Methods** | Single-centre parallel-group randomized controlled trial conducted in Germany from June to October 1999. |
| --- | --- |
| **Participants** | 54 participants aged 18 to 65 years old scheduled for magnetic resonance imaging (MRI) for the first time (mean age: 43 ± 14.6 years in the midazolam group and 49 ± 11.7 years in the placebo group; 48% men in midazolam group and 37% men in placebo group) |
| **Interventions** | 1. Intranasal midazolam 4 mg 2. Placebo |
| **Outcomes** | 1. Level of sedation measured 15 minutes after medication and after MRI. Participant sedation was evaluated by 1of the authors using a 5-point sedation scale (1 = agitated, non co-operative; 2 = alert, restless; 3 = calm, eyes spontaneously open; 4 = drowsy, responds to minor stimulation; 5 = asleep, rousable but does not respond to minor stimulation) 2. Numerical rating of anxiety measured 15 minutes after medication and after MRI. Visual Analogue Scale of Anxiety comprised an undivided 100-mm line, with 0 meaning “I am not anxious at all,” and 100 meaning “I am extremely anxious.” Participants were instructed to mark 1 point on the line that corresponded to the intensity of their anxiety at that moment 3. Incomplete procedures |
| **Identification** |  |
| **Notes** | Conflicts of interest or funding sources were not reported |

#### Risk of bias table

| **Bias** | **Authors' judgement** | **Support for judgement** |
| --- | --- | --- |
| Random sequence generation (selection bias) | Unclear risk | Not reported (not contacted for clarification) |
| Allocation concealment (selection bias) | Unclear risk | Not reported (not contacted for clarification) |
| Blinding of participants and personnel (performance bias) | Low risk | Blinded |
| Blinding of outcome assessment (detection bias) | Unclear risk | No information about blinding of assessor of sedation level. |
| Incomplete outcome data (attrition bias) | Low risk | No withdrawals |
| Selective reporting (reporting bias) | Unclear risk | No clear evidence that measured outcomes were not reported (trial protocols were not sought for confirmation) |
| Other bias | Low risk | None expected |

### Korttila 1985

| **Methods** | Single-centre parallel-group randomized controlled trial conducted in Finland |
| --- | --- |
| **Participants** | 76 adults undergoing rigid diagnostic bronchoscopy (25 participants randomized to 'low dose' midazolam were excluded from the review). Mean age: 55 ± 11 in the diazepam group and 55 ± 11 in the midazolam group. 70% men in the diazepam group and 58% men in the midazolam group |
| **Interventions** | **Intravenous administration of**   1. Midazolam 0.05 mg/kg (not used in the review) 2. Midazolam 0.1 mg/kg 3. Diazepam 0.2 mg/kg |
| **Outcomes** | **Measured during the procedure**   1. Participant and proceduralist satisfaction (0 = poor, 100 = good)   **Measured 2 hours after procedure**   1. Anterograde amnesia (recalled procedures) 2. Quality of recovery (could not walk in straight line at 2 hours after procedure) |
| **Identification** |  |
| **Notes** | Conflicts of interest or funding sources were not reported |

#### Risk of bias table

| **Bias** | **Authors' judgement** | **Support for judgement** |
| --- | --- | --- |
| Random sequence generation (selection bias) | Unclear risk | Not reported (not contacted for clarification) |
| Allocation concealment (selection bias) | Unclear risk | Not reported (not contacted for clarification) |
| Blinding of participants and personnel (performance bias) | Low risk | The participant and the bronchoscopist were unaware of the identity of the drug being administered. |
| Blinding of outcome assessment (detection bias) | Low risk | The investigator was unaware of the identity of the drug being administered. |
| Incomplete outcome data (attrition bias) | Low risk | No withdrawals |
| Selective reporting (reporting bias) | Unclear risk | No clear evidence that measured outcomes were not reported (trial protocols were not sought for confirmation) |
| Other bias | Low risk | None expected |

### Kuganeswaran 1999

| **Methods** | Single-centre parallel-group randomized controlled trial conducted in USA |
| --- | --- |
| **Participants** | 99 adults undergoing sigmoidoscopy (age 51 ± 2 years; 13 men and 35 women) |
| **Interventions** | 1. Oral midazolam 7.5 mg (participants were asked to swish medication in mouth 15 times before swallowing to allow improved absorption by the oral mucosa) 2. Placebo |
| **Outcomes** | **Measured during the procedure**   1. Level of sedation using a sedation assessment scale (scores ranged from 0 = awake to 3 = asleep, responsive only to direct verbal or physical stimulus) 2. Numerical rating of anxiety reported by proceduralist and participant (0 = no anxiety; 10 = severe anxiety) 3. Discomfort/pain reported by proceduralist and participant (0 = no pain; 10 = severe pain) 4. Incomplete procedures   **Measured after the procedure**   1. Participant satisfaction (refuse repeat procedure with same sedation) 2. Participant satisfaction (participant considered sedation was not adequate) 3. Anterograde amnesia (defined by number of participants who recalled the procedure) |
| **Identification** |  |
| **Notes** | Conflicts of interest were not reported but it was noted that Roche Pharmaceuticals provided the study medications and "funded in part" the study. The role of the funder in design, analysis or reporting was not disclosed |

#### Risk of bias table

| **Bias** | **Authors' judgement** | **Support for judgement** |
| --- | --- | --- |
| Random sequence generation (selection bias) | Low risk | Random-number table |
| Allocation concealment (selection bias) | Unclear risk | Not reported (not contacted for clarification) |
| Blinding of participants and personnel (performance bias) | Low risk | Double-blind - Quote: "the study medication was prepared by the inpatient pharmacy and physicians, nurses and patients were blinded to its identity" |
| Blinding of outcome assessment (detection bias) | Low risk | Double-blind - Quote: "the study medication was prepared by the inpatient pharmacy and physicians, nurses and patients were blinded to its identity" |
| Incomplete outcome data (attrition bias) | Low risk | 2 participants withdrew before receiving medication |
| Selective reporting (reporting bias) | Unclear risk | No clear evidence that measured outcomes were not reported (trial protocols were not sought for confirmation) |
| Other bias | Low risk | None expected |

### Lavies 1988

| **Methods** | Single-centre parallel group randomized controlled trial conducted in USA |
| --- | --- |
| **Participants** | 120 adults undergoing upper gastrointestinal endoscopy (mean age: 58 years in placebo group, 55 in diazepam group, 50 in midazolam group; 55% men in the placebo group, 70% men in the diazepam group, 48% men in the midazolam group) |
| **Interventions** | **Intravenous administration of**   1. Midazolam 2.5 - 7.5 mg 2. Diazepam 2.5 - 10 mg 3. Placebo |
| **Outcomes** | **Measured during the procedure**   1. Tolerance of procedure |
| **Identification** |  |
| **Notes** | Conflicts of interest or funding sources were not reported |

#### Risk of bias table

| **Bias** | **Authors' judgement** | **Support for judgement** |
| --- | --- | --- |
| Random sequence generation (selection bias) | Unclear risk | Random-number sequence used but Quote: "The study was continued until 40 patients were included in each group" |
| Allocation concealment (selection bias) | Unclear risk | Not reported (not contacted for clarification) |
| Blinding of participants and personnel (performance bias) | Low risk | Participant was unaware of medication |
| Blinding of outcome assessment (detection bias) | Unclear risk | Not reported (not contacted for clarification) |
| Incomplete outcome data (attrition bias) | Low risk | No withdrawals |
| Selective reporting (reporting bias) | Unclear risk | No clear evidence that measured outcomes were not reported (trial protocols were not sought for confirmation) |
| Other bias | Low risk | None expected |

### Lazaraki 2007

| **Methods** | Single-centre parallel-group randomized controlled trial conducted in Greece from July to December 2004. |
| --- | --- |
| **Participants** | 126 adults who were 23 to 84 years of age undergoing colonoscopy for the first time |
| **Interventions** | **Intravenous administration of**   1. 25 - 50 mcg fentanyl (mean 36 mcg) 2. 2 - 5 mg midazolam (mean 4.6 mg) |
| **Outcomes** | **Measured during the procedure**   1. Discomfort, pain (0 = very well/no discomfort, 4 = unbearable) 2. Incomplete procedures 3. Sedation reversal   **Measured after the procedure**   1. Participant satisfaction (measured in the trial as willingness to undergo another procedure with same medication) 2. Anterograde amnesia (recalled procedures) |
| **Identification** |  |
| **Notes** | Conflicts of interest or funding sources were not reported |

#### Risk of bias table

| **Bias** | **Authors' judgement** | **Support for judgement** |
| --- | --- | --- |
| Random sequence generation (selection bias) | Unclear risk | Not reported (not contacted for clarification) |
| Allocation concealment (selection bias) | Unclear risk | Not reported (not contacted for clarification) |
| Blinding of participants and personnel (performance bias) | Unclear risk | Not reported (not contacted for clarification) |
| Blinding of outcome assessment (detection bias) | Low risk | Monitoring was performed by a single specialist nurse blinded to the randomization |
| Incomplete outcome data (attrition bias) | Low risk | No withdrawals |
| Selective reporting (reporting bias) | Unclear risk | No clear evidence that measured outcomes were not reported (trial protocols were not sought for confirmation) |
| Other bias | Low risk | None expected |

### Lee 1989

| **Methods** | Single-centre parallel-group randomized controlled trial conducted in Jamaica |
| --- | --- |
| **Participants** | 149 adults undergoing upper GI endoscopy for the first time. 79 men with a mean age of 52.5 years (range 18 to 81) and 70 women with a mean age of 46 years (17 to 82 years) |
| **Interventions** | **Intravenous administration of**   1. Diazepam to a maximum of 0.15 mg/kg 2. Midazolam to a maximum of 0.07 mg/kg 3. No sedation (not used in this review) (47 participants) |
| **Outcomes** | **Measured during the procedure**   1. Participant co-operation 2. Difficulty performing procedure   **Measured 24 to 48 hours after the procedure**   1. Anterograde amnesia (defined by number of participants who recalled the procedure) 2. Discomfort/pain (measured in the trial as 'uncomfortable') |
| **Identification** |  |
| **Notes** | Conflicts of interest or funding sources were not reported |

#### Risk of bias table

| **Bias** | **Authors' judgement** | **Support for judgement** |
| --- | --- | --- |
| Random sequence generation (selection bias) | Unclear risk | Not reported (not contacted for clarification) |
| Allocation concealment (selection bias) | Unclear risk | Not reported (not contacted for clarification) |
| Blinding of participants and personnel (performance bias) | High risk | Not blinded |
| Blinding of outcome assessment (detection bias) | High risk | The endoscopist who was unaware of the drug used completed a questionnaire to assess the participant co-operation and difficulty performing procedure outcomes, but anterograde amnesia and discomfort/pain were not assessed in a blinded fashion (no information about participant blinding) |
| Incomplete outcome data (attrition bias) | Low risk | No withdrawals |
| Selective reporting (reporting bias) | Unclear risk | No clear evidence that measured outcomes were not reported (trial protocols were not sought for confirmation) |
| Other bias | Low risk | None expected |

### Manning 2016

| **Methods** | A parallel group randomized controlled trial in USA from June 2011 to May 2013. |
| --- | --- |
| **Participants** | **Baseline characteristics**   1. Intranasal midazolam:    1. Males (%): 70    2. Mean age: 40.2 2. Intranasal dexmedetomidine:    1. Males (%): 77    2. Mean age: 35 3. Overall:    1. Males (%): 74    2. Mean age: 37.3 (IQR 22,54)   **Inclusion criteria**(23 participants)   1. Between18 and 60 years of age 2. Haemodynamically stable 3. Undergoing NGT placement by a registered nurse (RN) for diagnostic or treatment purposes as part of their ongoing health care   **Exclusion criteria**   1. Pregnant 2. Incarcerated 3. Displayed an altered mental status 4. Had a body weight 40 kg 5. Had a known hypersensitivity to benzodiazepines 6. ad any other contraindications to benzodiazepine use (e.g., recent benzodiazepine use) 7. Unable to provide consent. (Research staff obtained written consent from both the patient and the nurse performing the placement prior to conducting the study.)   **Pretreatment**   1. More patients in control group had 16G (larger) NG tube than in treatment group |
| **Interventions** | **Intervention characteristics**   1. Midazolam 2 mg intravenous 2. Placebo |
| **Outcomes** | 1. **Pain**    1. Outcome type: continuous outcome    2. Reporting: fully reported    3. Scale: VAS    4. Range: 0 (none) to 100 (unbearable)    5. Unit of measure: mm    6. Direction: lower is better    7. Data value: Eendpoint 2. **Discomfort**    1. Outcome type: continuous outcome    2. Reporting: fully reported    3. Scale: mm    4. Range: 0 (none) to 100 (unbearable)    5. Unit of measure: VAS    6. Direction: lower is better    7. Data value: endpoint 3. **Remember procedure (anterograde amnesia)**    1. Outcome type: dichotomous outcome    2. Reporting: fully reported    3. Direction: lower is better    4. Data value: endpoint 4. **Discomfort**    1. Outcome type: dichotomous outcome    2. Reporting: fully reported    3. Unit of measure: answered agree strongly agree    4. Direction: lower is better    5. Data value: endpoint 5. **Anxiety**    1. Outcome type: dichotomous outcome    2. Reporting: fully reported    3. Unit of measure: answered agree or strongly agree    4. Direction: lower is better    5. Data value: endpoint 6. **Satisfaction**    1. Outcome type: dichotomous outcome    2. Reporting: fully reported    3. Unit of measure: answered agree or strongly agree    4. Direction: higher is better    5. Data value: endpoint 7. **Incomplete procedure**    1. Outcome type: dichotomous outcome    2. Reporting: fully reported    3. Direction: lower is better    4. Data value: endpoint 8. **Difficult to perform procedure**    1. Outcome type: dichotomous outcome    2. Reporting: fully reported    3. Direction: lower is better    4. Data value: endpoint |
| **Identification** |  |
| **Notes** | **Sponsorship source:** sSupported by Fletcher Allen Health Care.The authors have no potential conflicts to disclose.  **Country:** USA  **Setting:** eEmergency department  **Comments:** n/a  **Authors name:** Chelsea Taylor Manning  **Institution:** Department of Surgery, University of Vermont College of Medicine, Burlington, VT.  **Email:** Kalev Freeman; e-mail: kalev.freeman@uvm.edu.  **Address:** University of Vermont College of MedicineBurlington VT |

#### Risk of bias table

| **Bias** | **Authors' judgement** | **Support for judgement** |
| --- | --- | --- |
| Random sequence generation (selection bias) | Low risk | Quote: "The pharmacy randomized the kits in blocks of 4 using a table supplied by our statistician." |
| Allocation concealment (selection bias) | Low risk | Quote: "Providers, researchers, and subjects were blinded to kit contents." |
| Blinding of participants and personnel (performance bias) | Low risk | Quote: "The hospital pharmacy supplied 2 mg of midazolam or an equivalent volume of normal saline without preservatives in sequentially numbered kits that were identical in appearance, stored in a locked ED refrigerator, and replaced by pharmacy staff once each week during the study period." |
| Blinding of outcome assessment (detection bias) | Low risk | Quote: "safety by the research pharmacy. Nurses administered premedication with 1 mL of intranasal atomized cophenylcaine (lidocaine 5% with 0.5% phenylephrine) in each nostril, followed by the study drug (midazolam or control) given via slow IV injection (over the course of 2 minutes) during observation by research staff. The nurse then placed the room temperature NGT within 5 minutes of premedication. Research staff documented time of drug administration and insertion as well as nurse-obtained vital signs 5 minutes prior to, during, and 5 minutes after NGT insertion. Researchers attempted to interview the subjects 15 minutes after the procedure. If participants were unable to complete the survey, additional attempts were made until 45 minutes after the procedure. Both subjects and their nurses completed an interviewer- assisted questionnaire (Data Supplements S1 and S2, available as supporting information in the online version of this paper). Research staff recorded their observations in a standardized case-report form (Data Supplement S3, available as supporting information in the online version of this paper) and results were entered into REDCap. Measurements The primary outcome was" |
| Incomplete outcome data (attrition bias) | Low risk | Quote: "potentially eligible for the trial. Of the 23 subjects who were randomized to this pilot study, all 23 ﬁnished the study and provided complete data (Figure 1). The trial was suspended after" |
| Selective reporting (reporting bias) | Low risk | Quote: "This trial was registered through ClinicalTrials.gov under the identiﬁer NCT01375634 and the full protocol and original data may be obtained from the corresponding author. The"  Judgement Comment: Registered outcomes consistent with those reported. |
| Other bias | Low risk | Judgement Comment: None expected. |

### Mignonsin 1994

| **Methods** | Single-centre parallel group randomized controlled trial in France. |
| --- | --- |
| **Participants** | **Inclusion criteria** (100 participants)   1. Adullts ( > 15 years old) who underwent a gastroduodenal endoscopy for gastric pain or control of ulcer healing.   **Exclusion criteria**   1. Myasthenia gravis 2. Pregnancy 3. Past history of adverse reaction to benzodiazepines 4. Long term treatment with psychotropic medications |
| **Interventions** | **Intervention characteristics**  **Intravenous bolus administered once in 20 - 30 seconds of**   1. Intravenous midazolam 0.07 mg/kg ( < 65 years) or 0.06 mg/kg ( > 65 years), or 2. Intravenous diazepam 0.15 mg/kg ( < 65 years) or 0.11 mg/kg ( > 65 years) |
| **Outcomes** | 1. **Effective sedation**    1. Outcome type: dichotomous 2. **Retrograde and anterograde amnesia**    1. Outcome type: dichotomous 3. **Discomfort, pain**    1. Outcome type: dichotomous 4. **Disinhibition/excitation**    1. Outcome type: dichotomous |
| **Identification** |  |
| **Notes** | **Country:** France  **Setting:** Endoscopy  **Comments:** N/A  **Authors name:** Mignonsin D  **Sponsorship source: None** |

#### Risk of bias table

| **Bias** | **Authors' judgement** | **Support for judgement** |
| --- | --- | --- |
| Random sequence generation (selection bias) | High risk | No information provided about how the randomization was performed. |
| Allocation concealment (selection bias) | Unclear risk | No information provided about how the allocation sequence was concealed. |
| Blinding of participants and personnel (performance bias) | High risk | Unclear. Not blinded. |
| Blinding of outcome assessment (detection bias) | High risk | Not blinded. |
| Incomplete outcome data (attrition bias) | Low risk | No dropouts |
| Selective reporting (reporting bias) | Unclear risk | The trial was not registered. |
| Other bias | Low risk | None expected |

### Neville 2016

| **Methods** | A single-centre, double-blind, parallel group randomized controlled trial in the USA from July 1, 2014 to 5 March 5 2015. |
| --- | --- |
| **Participants** | **Baseline characteristics**   1. Intranasal midazolam:    1. Males (%): 67    2. Mean age: 3.15 2. Intranasal dexmedetomidine:    1. Males (%): 65    2. Mean age: 3.44 3. Overall:    1. Males (%): 66    2. Mean age: 3.30   **Inclusioncriteria** (38 participants)   1. Patients 1 - 5 years of age who presented to the paediatric ED with lacerations less than 5 cm that required suture repair with anxiolysis were eligible for enrolment.   **Exclusion criteria**   1. Laceration repair required intravenous sedation 2. Other injuries requiring attention 3. Allergy or sensitivity to midazolam or dexmedetomidine 4. Abnormal vital signs for age, particularly bradycardia or hypotension 5. Cardiac disease or Moya-Moya 6. An illness with significant nasal congestion |
| **Interventions** | Intranasal midazolam vs. intranasal dexmedetomidine |
| **Outcomes** | 1. **Anxiety during positioning for procedure**    1. Outcome type: continuous outcome    2. Scale: modified Yale Preoperative Anxiety Scale    3. Range: scores ranging from 23.3 to 100    4. Direction: higher scores indicate greater anxiety 2. **Number of participants who were not anxious during positioning for procedure**    1. Outcome type: dichotomous outcome    2. Scale: modified Yale Preoperative Anxiety Scale    3. Range: < 30    4. Direction: lower scores indicate less anxiety |
| **Identification** |  |
| **Notes** | **Sponsorship source:** University of Pittsburgh  **Country:** USA United States of America  **Setting:** Academic paediatric ED  **Comments:**  **Authors name:** Desiree Neville  **Institution:** Children's Hospital of Pittsburgh of University of Pittsburgh Medical Center  **Email:** desiree.neville@chp.edu  **Address:** Pediatric Emergency Medicine Fellow Physician, Children's Hospital of Pittsburgh of UPMC, Pittsburgh, Pennsylvania, USAnited States, 15224 |

#### Risk of bias table

| **Bias** | **Authors' judgement** | **Support for judgement** |
| --- | --- | --- |
| Random sequence generation (selection bias) | Low risk | Quote: "The research pharmacist, who was not otherwise involved in the study, generated a randomization schedule prior to initiation of the study. The randomization schedule was then used by the pharmacy to randomize the patients to receive either 0.4 mg/kg midazolam or 2 mg/kg dexmedetomidine. 3,8" |
| Allocation concealment (selection bias) | Low risk | Quote: "The randomization schedule that determined which medication was dispensed was not released to the research staff until completion of the study."  Quote: "Syringes sent from the pharmacy to the ED appeared the same and contained the same volume per syringe, regardless of medication." |
| Blinding of participants and personnel (performance bias) | Low risk | Quote: "All treating providers, nurses, patients, patient families, ancillary staff, researchers, and data analysts were blinded to the medication received." |
| Blinding of outcome assessment (detection bias) | Low risk | Quote: "care provider, not involved in scoring of the patient’s anxiety, administered the medication." |
| Incomplete outcome data (attrition bias) | Low risk | Quote: "There was one patient whose data were not collected due to equipment malfunction and they had no results to include in the analysis. Another patient received over twice his or her appropriate dose of medication due to a weight recording error and was withdrawn from the study and analysis. Both of these withdrawals occurred around the time of enrolment, before any unblinding or further patient enrolments." |
| Selective reporting (reporting bias) | Low risk | Quote: "The study was approved by the institutional review board at our institution and was registered as a clinical trial prior to initiation of the study (NCT02168439)." |
| Other bias | Low risk |  |

### Puttapitakpong 2015

| **Methods** | A parallel group randomized controlled trial in Thailand. |
| --- | --- |
| **Participants** | **Baseline Characteristics**   1. **Oral midazolam**    1. Males (%): 130    2. mean age: 53 ± 11 2. **Placebo**    1. Males (%): 130    2. mean age: 54 ± 12 3. **Overall**    1. Males (%): 55    2. mean age: 53.74 (11.74)   **Inclusion criteria** (260 participants)   1. Aged 18 to 70 who were scheduled to undergo an elective diagnostic EGD at the GI Endoscopy Unit, Phramongkutklao Hospital 2. Class 1 to 2 by the American Society of Anesthesiology (ASA) criteria   **Exclusion criteria**   1. History of gastrectomy, oesophagectomy, or pancreaticoduodenectomy 2. ASA class 3 to 4 3. History of allergy to midazolam 4. Alcoholism 5. Drug abuse 6. Psychotic drug ingestion within the preceding 3 months 7. Pregnancy   **Pretreatment**   1. Nil significant |
| **Interventions** | **Intervention characteristics**  **Oral midazolam**   1. Dose: 5 mg midazolam in 15 ml apple juice   **Placebo**   1. Dose: 15 ml apple juice |
| **Outcomes** | 1. **Discomfort**    1. Outcome type: continuous outcome 2. **Remember procedure (anterograde amnesia) Score of 3 or 4 (able to recall and describe most of or the entire procedure)**    1. Outcome type: dichotomous outcome 3. **Anxiety**    1. Outcome type: continuous outcome    2. Reporting: fully reported    3. Scale: visual analogue scale    4. Range: 0 - 10    5. Direction: lower is better    6. Data value: endpoint 4. **Satisfaction**    1. Outcome type: continuous outcome    2. Reporting: fully reported    3. Scale: VAS    4. Range: 0 - 10    5. Direction: higher is better    6. Data value: change from baseline 5. **Incomplete procedure**    1. Outcome type: dichotomous outcome 6. **Willingness to repeat procedure with same sedation**    1. Outcome type: dichotomous outcome 7. **Adverse event (aspiration)**    1. Outcome type: adverse event    2. Reporting: fully reported    3. Direction: lower is better    4. Data value: endpoint |
| **Identification** |  |
| **Notes** | **Sponsorship source:** tThe study was supported by Grant of the Gastroenterological Association of Thailand.Some midazolam was supplied by Pharma GmbH  **Country:** Thailand  **Setting:** Endoscopy  **Comments:**  **Authors name:** Krit Opuchar, M.D.,  **Institution:** Division of Gastroenterology, Department of Medicine, Phramongkutklao Hospital, Bangkok 10400, Thailand.  **Email:**  **Address:** Division of Gastroenterology, Department of Medicine, Phramongkutklao Hospital, Bangkok 10400, Thailand. |

#### Risk of bias table

| **Bias** | **Authors' judgement** | **Support for judgement** |
| --- | --- | --- |
| Random sequence generation (selection bias) | Low risk | Quote: "Patients were randomized by the computer to receive either 5 mg of midazolam hydrochloride mixed with 15 cc of apple juice or placebo (15 cc. of apple juice), given 30 minutes before EGD" |
| Allocation concealment (selection bias) | Unclear risk | Judgement comment: no information provided (not contacted for clarification) |
| Blinding of participants and personnel (performance bias) | Low risk | Quote: "All personnel in the study, including the, nurses and endoscopists as well as the patients, were blinded to the treatment modality" |
| Blinding of outcome assessment (detection bias) | Low risk | Judgement comment: data collected by nurses who were blinded to treatment allocation |
| Incomplete outcome data (attrition bias) | Low risk | Judgement comment: no attrition reported |
| Selective reporting (reporting bias) | Low risk | Judgement comment: all the outcomes in the methods section have results presented |
| Other bias | Low risk | Judgement comment: none expected |

### Rolo 2012

| **Methods** | Multicentre (2 sites) parallel-group randomized controlled trial conducted in Portugal from April to June 2009. |
| --- | --- |
| **Participants** | 100 adults undergoing fibreoptic bronchoscopy (mean age was 56 ± 14 years (range 18 - 79 years); 66% were men |
| **Interventions** | 1. 0.05 mg/kg intravenous midazolam 2. Placebo |
| **Outcomes** | **Measured during the procedure:**   1. Disinhibition/excitation (measured in the trial as 'agitation') 2. Sedation reversal   **Measured 1 hour after the procedure**   1. Participant satisfaction (willingness to undergo another procedure with the same medication) |
| **Identification** |  |
| **Notes** | Conflicts of interest or funding sources were not reported |

#### Risk of bias table

| **Bias** | **Authors' judgement** | **Support for judgement** |
| --- | --- | --- |
| Random sequence generation (selection bias) | Unclear risk | Not reported (not contacted for clarification) |
| Allocation concealment (selection bias) | Unclear risk | Not reported (not contacted for clarification) |
| Blinding of participants and personnel (performance bias) | Unclear risk | Did state it was double-blind but no specific information provided about how this was achieved |
| Blinding of outcome assessment (detection bias) | Unclear risk | Not reported (not contacted for clarification) |
| Incomplete outcome data (attrition bias) | Low risk | No withdrawals |
| Selective reporting (reporting bias) | Unclear risk | No clear evidence that measured outcomes were not reported (trial protocols were not sought for confirmation) |
| Other bias | Low risk | None expected |

### Sainpy 1984

| **Methods** | Parallel-group single-centre randomized controlled trial conducted in France |
| --- | --- |
| **Participants** | 64 adults who underwent a gastroduodenal endoscopy.  Exclusion of myasthenia gravis, pregnancy, past history of adverse reaction to benzodiazepines, long-term treatment with psychotropic medications |
| **Interventions** | 1. Intravenous midazolam: 0.1 mg under 65 yrs and 0.085 mg over 65 yrs infused in 30 seconds 2. Intravenous diazepam: 0.2 mg under 65 yrs and 0.15 mg over 65 yrs infused in 30 seconds |
| **Outcomes** | **Measured during the procedure**   1. Discomfort/pain (as defined/measured by the authors of the trial)   **Measured after the procedure**   1. Anterograde amnesia (defined by number of participants who recalled the procedure) |
| **Identification** |  |
| **Notes** | Conflicts of interest or funding sources were not reported |

#### Risk of bias table

| **Bias** | **Authors' judgement** | **Support for judgement** |
| --- | --- | --- |
| Random sequence generation (selection bias) | Unclear risk | Method not stated (not contacted for clarification) |
| Allocation concealment (selection bias) | Low risk | Used sealed envelopes |
| Blinding of participants and personnel (performance bias) | Unclear risk | The gastroenterologist was the only person blinded to sedative drug |
| Blinding of outcome assessment (detection bias) | Unclear risk | The gastroenterologist was the only person blinded to sedative drug |
| Incomplete outcome data (attrition bias) | Low risk | No withdrawals |
| Selective reporting (reporting bias) | Unclear risk | No clear evidence that measured outcomes were not reported (trial protocols were not sought for confirmation) |
| Other bias | Low risk | None expected |

### Salehi 2017

| **Methods** | A parallel group randomized controlled trial in Iran in 2015. |
| --- | --- |
| **Participants** | **Baseline characteristics**   1. **Midazolam 2 mg/kg oral**    1. Males (%): 21 (61.8)    2. mean age: 15.15 (18.92) months 2. **Chloral hydrate 50 mg/kg**    1. Males (%): 22 (64.7)    2. mean age: 14.28 (14.25) months 3. **Overall**    1. Males (%):    2. mean age:   **Inclusion criteria** (68 participants)   1. Children under 10 years for echocardiography who were fully consciousness 2. Lack of respiratory distress 3. Lack of hypotonia   **Exclusion criteria**   1. Not completing echocardiography due to restlessness of the child 2. Lack of parents’ cooperation because of the drug’s side effects, 3. Instability of patient’s vital signs before receiving the drug 4. Requiring intensive care unit care   **Pretreatment**   1. No differences between groups with regard to age, gender |
| **Interventions** | **Intervention characteristics**   1. Midazolam 2 mg/kg oral 2. Chloral hydrate 50 mg/kg |
| **Outcomes** | 1. **Incomplete procedure**    1. Outcome type: dichotomous outcome 2. **Moderate sedation**    1. Outcome type: dichotomous outcome    2. Reporting: partially reported    3. Scale: RASS    4. Data value: endpoint |
| **Identification** |  |
| **Notes** | **Sponsorship source:** fFinancial support was provided by the Vice Chancellor for Research and Technology of Birjand University of Medical Sciences.  **Country:** Iran  **Setting:** Birjand city’s Valiasr Pediatric Hospital and Heart Clinic  **Comments:**  **Authors name:** Ali Ebrahimzadeh  **Institution:** Birjand University of Medical Sciences  **Email:** Ebrahimzadehali@yahoo.com  **Address:** Ghafari Avenue, Birjand, South Khorasan 9717853577, Iran |

#### Risk of bias table

| **Bias** | **Authors' judgement** | **Support for judgement** |
| --- | --- | --- |
| Random sequence generation (selection bias) | Unclear risk | Quote: "Patients were randomly divided into 2 therapeutic groups using 1:1 blocks." |
| Allocation concealment (selection bias) | Unclear risk | Not reported (not contacted for clarification) |
| Blinding of participants and personnel (performance bias) | Unclear risk | Quote: "Oral midazolam (2.0 mg/kg) or oral chloral hydrate syrup (50 mg/kg) was indicated for the first and second groups, respectively (the dose of these 2 drugs was determined according to the latest articles on paediatric sedation before noninvasive procedures; a 10% concentration of the chloral hydrate syrup was provided by Birjand’s Valiasr Hospital pharmacy, and for oral midazolam the Darou Pakhsh 5 mg/mL midazolam syrup was used)."  Judgement Comment: no statement about how blinding was achieved |
| Blinding of outcome assessment (detection bias) | Low risk | Judgement comment: stated it was 'double-blind' (patient and observer) |
| Incomplete outcome data (attrition bias) | High risk | Judgement comment: patients were excluded if the child was too restless for echocardiography to be completed. |
| Selective reporting (reporting bias) | Unclear risk | Judgement comment: trial was not registered |
| Other bias | Low risk | Judgement comment: none expected |

### Stokland 2003

| **Methods** | Single-centre parallel-group randomized controlled trial conducted in Sweden |
| --- | --- |
| **Participants** | 95 children referred for voiding cystourethrography (median age 2.2 years in midazolam group and 3.2 years in placebo group) |
| **Interventions** | 1. Intranasal midazolam given in a dose of 0.2 mg/kg body weight with a maximum dose of 5 mg 2. Placebo group was given saline 0.9 mg/ml sterile solution |
| **Outcomes** | Incomplete procedures |
| **Identification** |  |
| **Notes** | Conflicts of interest or funding sources were not reported |

#### Risk of bias table

| **Bias** | **Authors' judgement** | **Support for judgement** |
| --- | --- | --- |
| Random sequence generation (selection bias) | Unclear risk | Not reported (not contacted for clarification) |
| Allocation concealment (selection bias) | Low risk | The children were stratified by gender and randomly allocated to 1 of 2 groups, midazolam or placebo, by opening a sealed envelope prepared in blocks of 4 |
| Blinding of participants and personnel (performance bias) | Low risk | The substances were available in bottles with serial numbers, but were otherwise of identical appearance |
| Blinding of outcome assessment (detection bias) | Unclear risk | Not reported (not contacted for clarification) |
| Incomplete outcome data (attrition bias) | Low risk | No withdrawals |
| Selective reporting (reporting bias) | Unclear risk | No clear evidence that measured outcomes were not reported (trial protocols were not sought for confirmation) |
| Other bias | Low risk | None expected |

### Takrouri 1988

| **Methods** | Single-centre parallel-group randomized controlled trial conducted in Jordan |
| --- | --- |
| **Participants** | 120 adults undergoing endoscopy (mean age 39 ± 0.6 in diazepam group, 41 ± 0.9 in flunitrazepam group and 34 ± 0.6 in midazolam group) |
| **Interventions** | Mean doses: diazepam 5 mg; flunitrazepam 0.65 mg; midazolam 5.8 mg (all intravenous) |
| **Outcomes** | **Measured during the procedure**  (as defined/measured by the authors of the trial)   1. Number of participants rated as 'anxious' 2. Difficulty performing procedure (rated as difficult to perform procedure) 3. Participant co-operation (not co-operative)   **Measured after the procedure (before discharge)**   1. Anterograde amnesia (defined by number of participants who recalled the procedure) 2. Quality of recovery (rated as delayed recovery) |
| **Identification** |  |
| **Notes** | Conflicts of interest or funding sources were not reported |

#### Risk of bias table

| **Bias** | **Authors' judgement** | **Support for judgement** |
| --- | --- | --- |
| Random sequence generation (selection bias) | Low risk | Random-number table |
| Allocation concealment (selection bias) | Unclear risk | Not reported (not contacted for clarification) |
| Blinding of participants and personnel (performance bias) | High risk | Not blinded |
| Blinding of outcome assessment (detection bias) | High risk | Not blinded |
| Incomplete outcome data (attrition bias) | Low risk | No withdrawals reported |
| Selective reporting (reporting bias) | Unclear risk | No clear evidence that measured outcomes were not reported (trial protocols were not sought for confirmation) |
| Other bias | Low risk | None expected |

### Templeton 2010

| **Methods** | Single-centre parallel-group randomized controlled trial conducted in UK from April 2003 to August 2006. |
| --- | --- |
| **Participants** | 42 children requiring removal of Kirschner wires in an orthopaedic outpatient department (average age 7.1 years; range 3.6 to 12.3 years) |
| **Interventions** | 1. 0.2 mL/kg of 1 mg/mL oral midazolam 2. Placebo |
| **Outcomes** | Numerical rating of anxiety |
| **Identification** |  |
| **Notes** | Funding source was reported (research grant from Beatrice Jennings Trust Fund at The General Infirmary at Leeds) |

#### Risk of bias table

| **Bias** | **Authors' judgement** | **Support for judgement** |
| --- | --- | --- |
| Random sequence generation (selection bias) | Low risk | Randomization was performed by the pharmacy department using random-number tables |
| Allocation concealment (selection bias) | Low risk | Sealed envelopes each containing a code number |
| Blinding of participants and personnel (performance bias) | Low risk | Corresponding coded bottles contained either midazolam 1.0 mg/mL or the placebo |
| Blinding of outcome assessment (detection bias) | Unclear risk | Not reported (not contacted for clarification) |
| Incomplete outcome data (attrition bias) | High risk | Intention-to-treat analysis not used |
| Selective reporting (reporting bias) | Unclear risk | No clear evidence that measured outcomes were not reported (trial protocols were not sought for confirmation) |
| Other bias | Low risk | None expected |

### Tolia 1990

| **Methods** | Single-centre parallel-group randomized controlled trial conducted in USA |
| --- | --- |
| **Participants** | 41 children undergoing upper GI endoscopy |
| **Interventions** | **Intravenous administration of**   1. 0.1 - 0.15 mg/kg midazolam 2. 0.2 - 0.4 mg/kg diazepam |
| **Outcomes** | **Measured during procedures**   1. Participant co-operation   **Measured 24 hours after procedures**   1. Anterograde amnesia (recalled procedures) 2. Discomfort, pain |
| **Identification** |  |
| **Notes** | Conflicts of interest or funding sources were not reported |

#### Risk of bias table

| **Bias** | **Authors' judgement** | **Support for judgement** |
| --- | --- | --- |
| Random sequence generation (selection bias) | Unclear risk | Not reported (not contacted for clarification) |
| Allocation concealment (selection bias) | Unclear risk | Not reported (not contacted for clarification) |
| Blinding of participants and personnel (performance bias) | Low risk | Both endoscopist and participant were blinded |
| Blinding of outcome assessment (detection bias) | Low risk | Both endoscopist and participant were blinded |
| Incomplete outcome data (attrition bias) | High risk | Not analysed as intention-to-treat |
| Selective reporting (reporting bias) | Unclear risk | No clear evidence that measured outcomes were not reported (trial protocols were not sought for confirmation) |
| Other bias | Low risk | None expected |

### Wheeler 2001

| **Methods** | Single-centre parallel-group randomized controlled trial conducted in USA from April 1997 to September 1998. |
| --- | --- |
| **Participants** | 40 children < 5 years of age undergoing echocardiography (13 boys (52%) in midazolam group and 10 boys (66%) in chloral hydrate group) |
| **Interventions** | 1. 75 mg/kg oral chloral hydrate 2. 0.5 mg/kg oral midazolam   Children requiring further sedation (as determined by the assigned nurse) received a second dose of the same medication 30 minutes after the initial dose, either 25 mg/kg chloral hydrate or 0.25 mg/kg midazolam |
| **Outcomes** | **Measured during the procedure**  (as defined/measured by the authors of the trial)   1. Incomplete procedures 2. Disinhibition/excitation |
| **Identification** |  |
| **Notes** | Conflicts of interest or funding sources were not reported |

#### Risk of bias table

| **Bias** | **Authors' judgement** | **Support for judgement** |
| --- | --- | --- |
| Random sequence generation (selection bias) | Low risk | Random-number table |
| Allocation concealment (selection bias) | Unclear risk | Not reported (not contacted for clarification) |
| Blinding of participants and personnel (performance bias) | Low risk | Echocardiographer blinded |
| Blinding of outcome assessment (detection bias) | Unclear risk | No information about blinding of outcome assessor |
| Incomplete outcome data (attrition bias) | Low risk | No withdrawals |
| Selective reporting (reporting bias) | Unclear risk | No clear evidence that measured outcomes were not reported (trial protocols were not sought for confirmation) |
| Other bias | Low risk | None expected |

### Whitwam 1983

| **Methods** | Single-centre parallel-group randomized controlled trial conducted in the United Kingdom |
| --- | --- |
| **Participants** | 100 adult ASA I - II participants undergoing upper GI endoscopy (29 men (58%) in midazolam group and 30 men (60%) in diazepam group; mean age 42 ± 19.9 in midazolam group and 44 ± 18.5 in diazepam group) |
| **Interventions** | **Intravenous administration of**   1. midazolam 0.07 mg/kg 2. diazepam 0.15 mg/kg |
| **Outcomes** | **Measured during the procedure**   1. Number of participants rated as 'anxious' 2. Difficulty performing procedure 3. Participant co-operation   **Measured on discharge from recovery area**   1. Anterograde amnesia (recalled procedures)   **Measured after the procedure (questionnaire sent to participants - no timeframe reported)**   1. Quality of recovery |
| **Identification** |  |
| **Notes** | Reported that 2 of the authors were supported by Roche Inc. |

#### Risk of bias table

| **Bias** | **Authors' judgement** | **Support for judgement** |
| --- | --- | --- |
| Random sequence generation (selection bias) | Unclear risk | Not reported (not contacted for clarification) |
| Allocation concealment (selection bias) | Unclear risk | Not reported (not contacted for clarification) |
| Blinding of participants and personnel (performance bias) | Unclear risk | Not reported (not contacted for clarification) |
| Blinding of outcome assessment (detection bias) | Low risk | Blind to investigators assessing participants |
| Incomplete outcome data (attrition bias) | Low risk | No withdrawals |
| Selective reporting (reporting bias) | Unclear risk | No clear evidence that measured outcomes were not reported (trial protocols were not sought for confirmation) |
| Other bias | Low risk | None expected |

### Yamasaki 2017

| **Methods** | A parallel group randomized controlled trial in Japan from March to May 2015. |
| --- | --- |
| **Participants** | **Baseline characteristics**  **Intravenous midazolam**   1. Males (%): 82.5 2. mean age: 72   **Pethidine**   1. Males (%): 84.6 2. mean age: 70   **Overall**   1. Males (%): 80.9 2. mean age: 71   **Inclusion criteria:** (120 participants)   1. Patients who had oesophageal squamous cell carcinoma (ESCC) before or under treatment 2. Patients who had a history of ESCC   **Exclusion criteria**   1. Pharyngeal cancer that was diagnosed before this study 2. A bleeding tendency 3. Severe organ failure   **Pretreatment**   1. Participants were allocated into three groups according to sedation method:    1. no sedation    2. midazolam group, or    3. pethidine group |
| **Interventions** | **Intervention characteristics**  **Intravenous midazolam**   1. Dose: 0.5 - 1 mg increments until eyes closed   **Pethidine**   1. Dose: 35 mg |
| **Outcomes** | 1. **Pain**    1. Outcome type: continuous outcome    2. Scale: VAS    3. Range: 0 = painless, 100 = extremely painful |
| **Identification** |  |
| **Notes** | **Sponsorship source:** Osaka Medical Center for Cancer and Cardiovascular Diseases  **Country:** Japan  **Setting:** Gastointestinal Oncology  **Comments:**  **Authors name:** Yasushi Yamasaki  **Institution:** Osaka Medical Center for Cancer and Cardiovascular Diseases  **Email:** yasshifive@yahoo.co.jp  **Address:** Department of Gastrointestinal Oncology, Osaka Medical Center for Cancer and Cardiovascular Diseases, 1-3-3, Higashinari-ku, Osaka 537-8511, Japan |

#### Risk of bias table

| **Bias** | **Authors' judgement** | **Support for judgement** |
| --- | --- | --- |
| Random sequence generation (selection bias) | Low risk |  |
| Allocation concealment (selection bias) | Low risk | Quote: "of sedation or no sedation). After stratiﬁcation, participants were randomly allocated into each of three groups according to the sedation method (no sedation group, midazolam group, or pethidine group). The sequence was concealed from the endoscopists until UGIE was assigned. Preparation and sedation for UGIE" |
| Blinding of participants and personnel (performance bias) | High risk | Quote: "it was a single-center and unblinded study." |
| Blinding of outcome assessment (detection bias) | High risk | Judgement comment: not blinded |
| Incomplete outcome data (attrition bias) | Low risk | Judgement comment: no missing data or dropouts |
| Selective reporting (reporting bias) | Low risk | Judgement comment: reported outcomes seem to match outcomes that were listed in study protocol (UMIN000016722)https://upload.umin.ac.jp/cgi-open-bin/ctr_e/ctr_view.cgi?recptno=R000019406 |
| Other bias | Low risk | Judgement comment: none identified |

### Younge 2001

| **Methods** | Single-centre parallel-group randomized controlled trial conducted in the UK |
| --- | --- |
| **Participants** | 59 children ages 1 to 7 needing laceration repair (mean age 4.1 years in both groups; 54% boys in midazolam group and 53% boys in ketamine group) |
| **Interventions** | 1. Oral midazolam 0.7 mg/kg 2. Oral ketamine 10 mg/kg |
| **Outcomes** | **Measured during the procedure**   1. Disinhibition/excitation |
| **Identification** |  |
| **Notes** | Reported that there were no conflicts of interest or funding received for the trial |

#### Risk of bias table

| **Bias** | **Authors' judgement** | **Support for judgement** |
| --- | --- | --- |
| Random sequence generation (selection bias) | Low risk | Drug solutions were pre-prepared by the hospital pharmacy and numbered sequentially, randomly containing 1 or other drug |
| Allocation concealment (selection bias) | Low risk | Drug solutions were pre-prepared by the hospital pharmacy and numbered sequentially, randomly containing 1 or other drug |
| Blinding of participants and personnel (performance bias) | Low risk | Double-blind |
| Blinding of outcome assessment (detection bias) | Low risk | Double-blind |
| Incomplete outcome data (attrition bias) | Low risk | No withdrawals |
| Selective reporting (reporting bias) | Unclear risk | No clear evidence that measured outcomes were not reported (trial protocols were not sought for confirmation) |
| Other bias | Low risk | None expected |

### Yuno 1996

| **Methods** | Single-centre parallel-group randomized controlled trial conducted in Japan |
| --- | --- |
| **Participants** | 40 adults undergoing colonoscopy for polyp removal |
| **Interventions** | 1. 0.05 mg/kg intravenous midazolam 2. Placebo |
| **Outcomes** | **Measured during the procedure**  *(as defined/measured by the authors of the trial)*   1. Participant satisfaction (measured in the trial as participant-assessed adequacy of sedation) 2. Proceduralist satisfaction (measured in the trial as proceduralist-assessed adequacy of sedation) |
| **Identification** |  |
| **Notes** | Reported that a grant from the Osaka Association for Prevention of Adult Diseases funded the study. No conflicts of interest were reported |

#### Risk of bias table

| **Bias** | **Authors' judgement** | **Support for judgement** |
| --- | --- | --- |
| Random sequence generation (selection bias) | Unclear risk | Not reported (not contacted for clarification) |
| Allocation concealment (selection bias) | Low risk | Quote. "Envelope method" |
| Blinding of participants and personnel (performance bias) | Unclear risk | Noted it was double-blind but no specific information provided |
| Blinding of outcome assessment (detection bias) | Unclear risk | Noted it was double-blind but no specific information provided |
| Incomplete outcome data (attrition bias) | Low risk | No withdrawals |
| Selective reporting (reporting bias) | Unclear risk | No clear evidence that measured outcomes were not reported (trial protocols were not sought for confirmation) |
| Other bias | Low risk |  |

###### Footnotes

ASA: American Society of Anesthesiology
BPM: beats per minute

ED: emergency department

EGD: enterogastroduodenal endoscopy

GI: gastrointestinal

HR: heart rate

IQR: interquartile range

IV: intravenous

MAP: mean arterial pressure

MRI: magnetic resonance Imaging

NGT: nasogastric tube

RASS: Richmod Agitation Sedation Scale

RCT: randomized controlled trial

RN: Registered Nurse

SBP: systolic blood pressure

VAS: visual analogue scale

vs: versus

yrs: years
