# Supplementary material for: Midazolam for sedation before procedures in adults and children: a systematic review update
Source: Syst Rev. 2021 Mar 5;10:69. doi: 10.1186/s13643-021-01617-5 (PMC7936483; doi:10.1186/s13643-021-01617-5)
Supplement: Supplementary file 4 — Additional file 4. Results: Expanded description of results for all comparisons included in the updated review [file 13643_2021_1617_MOESM4_ESM.docx]

# Results

## Description of studies

### Results of the search

[Figure 1](#FIG-01) summarizes the search results to December 2018. The searches identified 5215 potential studies. We retrieved 185 papers for consideration and included 38 trials in this review.

### Included studies

We included eight new trials in this updated review ([Manning 2016](#STD-Manning-2016); [Mignonsin 1994](#STD-Mignonsin-1994); [Neville 2016](#STD-Neville-2016); [Puttapitakpong 2015](#STD-Puttapitakpong-2015); [Salehi 2017](#STD-Salehi-2017); [Yamasaki 2017](#STD-Yamasaki-2017)).

In total, we included 38 trials with 3344 participants ([Characteristics of included studies](#CHARACTERISTICS_OF_INCLUDED_STUDIES)), that compared pre-procedure midazolam via the intravenous, oral and intranasal routes of administration, to either a placebo or alternative anxiolytic. The included trials were conducted in both adult and paediatric populations. Nineteen trials enrolled participants having gastrointestinal endoscopy procedures ([Bell 1988](#STD-Bell-1988); [Bhalla 2006](#STD-Bhalla-2006); [Bianchi Porro 1988](#STD-Bianchi-Porro-1988); [Cole 1983](#STD-Cole-1983); [Córdova 1992](#STD-C_x00f3_rdova-1992); [Fakheri 2010](#STD-Fakheri-2010); [Gilvarry 1990](#STD-Gilvarry-1990); [Kuganeswaran 1999](#STD-Kuganeswaran-1999); [Lavies 1988](#STD-Lavies-1988); [Lazaraki 2007](#STD-Lazaraki-2007); [Lee 1989](#STD-Lee-1989); [Mignonsin 1994](#STD-Mignonsin-1994); [Puttapitakpong 2015](#STD-Puttapitakpong-2015); [Sainpy 1984](#STD-Sainpy-1984); [Takrouri 1988](#STD-Takrouri-1988); [Tolia 1990](#STD-Tolia-1990); [Whitwam 1983](#STD-Whitwam-1983); [Yamasaki 2017](#STD-Yamasaki-2017); [Yuno 1996](#STD-Yuno-1996)), and there were three trials involving bronchoscopy ([Aktogu 1994](#STD-Aktogu-1994); [Korttila 1985](#STD-Korttila-1985); [Rolo 2012](#STD-Rolo-2012)). Diagnostic imaging was performed in seven trials ([Akil 2005](#STD-Akil-2005); [Alp 2019](#STD-Alp-2019); [D'Agostino 2000](#STD-D_x0027_Agostino-2000); [Hollenhorst 2001](#STD-Hollenhorst-2001); [Salehi 2017](#STD-Salehi-2017); [Stokland 2003](#STD-Stokland-2003); [Wheeler 2001](#STD-Wheeler-2001)). One trial was conducted with participants undergoing cardioversion ([Coll-Vinent 2003](#STD-Coll_x002d_Vinent-2003)). One trial was conducted with participants requiring nasogastric tube insertion in the emergency department ([Manning 2016](#STD-Manning-2016)). One trial was conducted with participants undergoing minor office-based plastic surgery ([De Alencar 2010](#STD-De-Alencar-2010)). One trial was conducted with children undergoing lumbar puncture ([Derakhshanfar 2013](#STD-Derakhshanfar-2013)). Three trials were conducted with children requiring suturing ([Everitt 2002](#STD-Everitt-2002); [Neville 2016](#STD-Neville-2016); [Younge 2001](#STD-Younge-2001)). One trial was conducted with children requiring Kirschner wire removal ([Templeton 2010](#STD-Templeton-2010)). One trial was conducted with children undergoing Intussusception Hydrostatic Reduction (Eissapur 2015). In total, 13 of the trials were conducted with children ([Salehi 2017](#STD-Salehi-2017); [Akil 2005](#STD-Akil-2005); [Alp 2019](#STD-Alp-2019);  [D'Agostino 2000](#STD-D_x0027_Agostino-2000); [Derakhshanfar 2013](#STD-Derakhshanfar-2013); [Eisapour 2015](#STD-Eisapour-2015); [Everitt 2002](#STD-Everitt-2002); [Neville 2016](#STD-Neville-2016); [Younge 2001](#STD-Younge-2001); [Stokland 2003](#STD-Stokland-2003); [Templeton 2010](#STD-Templeton-2010); [Tolia 1990](#STD-Tolia-1990); [Wheeler 2001](#STD-Wheeler-2001); ).

#### Settings

There was geographic variability across the included trials. Trials were performed in Australia ([Everitt 2002](#STD-Everitt-2002)), Brazil ([De Alencar 2010](#STD-De-Alencar-2010)), France ([Mignonsin 1994](#STD-Mignonsin-1994); [Sainpy 1984](#STD-Sainpy-1984)), Finland ([Korttila 1985](#STD-Korttila-1985)), Germany ([Hollenhorst 2001](#STD-Hollenhorst-2001)), Greece ([Lazaraki 2007](#STD-Lazaraki-2007)), India ([Bhalla 2006](#STD-Bhalla-2006)), Iran ([Derakhshanfar 2013](#STD-Derakhshanfar-2013); [Eisapour 2015](#STD-Eisapour-2015); [Fakheri 2010](#STD-Fakheri-2010); [Salehi 2017](#STD-Salehi-2017)), Italy ([Bianchi Porro 1988](#STD-Bianchi-Porro-1988)), Japan ([Yamasaki 2017](#STD-Yamasaki-2017); [Yuno 1996](#STD-Yuno-1996)), Jamaica ([Lee 1989](#STD-Lee-1989)), Jordan ([Takrouri 1988](#STD-Takrouri-1988)), Mexico ([Córdova 1992](#STD-C_x00f3_rdova-1992)), Portugal ([Rolo 2012](#STD-Rolo-2012)), Spain ([Coll-Vinent 2003](#STD-Coll_x002d_Vinent-2003)), Sweden ([Stokland 2003](#STD-Stokland-2003)), Thailand ) ([Puttapitakpong 2015](#STD-Puttapitakpong-2015)), Turkey ([Akil 2005](#STD-Akil-2005); [Alp 2019](#STD-Alp-2019); [Aktogu 1994](#STD-Aktogu-1994)), UK ([Bell 1988](#STD-Bell-1988); [Gilvarry 1990](#STD-Gilvarry-1990); [Templeton 2010](#STD-Templeton-2010); [Whitwam 1983](#STD-Whitwam-1983); [Younge 2001](#STD-Younge-2001)) and USA ([Cole 1983](#STD-Cole-1983); [D'Agostino 2000](#STD-D_x0027_Agostino-2000); [Kuganeswaran 1999](#STD-Kuganeswaran-1999); [Lavies 1988](#STD-Lavies-1988); [Manning 2016](#STD-Manning-2016); [Neville 2016](#STD-Neville-2016); [Tolia 1990](#STD-Tolia-1990); [Wheeler 2001](#STD-Wheeler-2001)).

#### Characteristics of interventions

Regarding the characteristics of the interventions, for intravenous midazolam, five trials used weight-based calculation with a dose of 0.1 mg/kg ([Córdova 1992](#STD-C_x00f3_rdova-1992); [Eisapour 2015](#STD-Eisapour-2015); [Korttila 1985](#STD-Korttila-1985); [Sainpy 1984](#STD-Sainpy-1984); [Tolia 1990](#STD-Tolia-1990)). Other trials used smaller doses, including 0.07 mg/kg ([Bianchi Porro 1988](#STD-Bianchi-Porro-1988); [Lee 1989](#STD-Lee-1989); [Mignonsin 1994](#STD-Mignonsin-1994); [Whitwam 1983](#STD-Whitwam-1983)), 0.06 mg/kg ([Aktogu 1994](#STD-Aktogu-1994); [Mignonsin 1994](#STD-Mignonsin-1994)) and 0.05 mg/kg ([Rolo 2012](#STD-Rolo-2012); [Yuno 1996](#STD-Yuno-1996)). One trial used a higher dose of 0.2 mg/kg ([Coll-Vinent 2003](#STD-Coll_x002d_Vinent-2003)). Other trials did not use participants' weight to calculate doses. These trials used either 2 mg ([Manning 2016](#STD-Manning-2016)), 2.5 mg ([Bell 1988](#STD-Bell-1988)), 5 mg ([Bhalla 2006](#STD-Bhalla-2006); [Cole 1983](#STD-Cole-1983)), 10 mg ([Gilvarry 1990](#STD-Gilvarry-1990)), or 15 mg ([De Alencar 2010](#STD-De-Alencar-2010)). Some trials used smaller doses for elderly participants ([Bell 1988](#STD-Bell-1988); [Bhalla 2006](#STD-Bhalla-2006); [Cole 1983](#STD-Cole-1983)). Other trials reported only the mean or range of dose that was administered instead of the planned method of titration ([Fakheri 2010](#STD-Fakheri-2010); [Lavies 1988](#STD-Lavies-1988); [Lazaraki 2007](#STD-Lazaraki-2007); [Takrouri 1988](#STD-Takrouri-1988); [Yamasaki 2017](#STD-Yamasaki-2017)). For oral midazolam, two trials did not use a weight-based dose calculation ([Kuganeswaran 1999](#STD-Kuganeswaran-1999); [Puttapitakpong 2015](#STD-Puttapitakpong-2015)). Weight-based doses of oral midazolam used were 0.5 mg/kg ([D'Agostino 2000](#STD-D_x0027_Agostino-2000); [Derakhshanfar 2013](#STD-Derakhshanfar-2013); [Wheeler 2001](#STD-Wheeler-2001)), 0.6 mg/kg ([Akil 2005](#STD-Akil-2005)), 0.7 mg/kg ([Younge 2001](#STD-Younge-2001)), 1 mg/kg ([Everitt 2002](#STD-Everitt-2002); [Templeton 2010](#STD-Templeton-2010)), and 2 mg/kg ([Salehi 2017](#STD-Salehi-2017)). For intranasal midazolam, [Hollenhorst 2001](#STD-Hollenhorst-2001) used a standard dose of 4 mg whereas [Stokland 2003](#STD-Stokland-2003) used a dose of 0.2 mg/kg up to 5 mg,  [Neville 2016](#STD-Neville-2016) used a dose of 0.4 mg/kg, and [Alp 2019](#STD-Alp-2019) used a dose of 0.2m/kg for their trial with children.

#### Comparator arms

The comparator arms were: intravenous diazepam in 15 trials with 1169 participants ([Aktogu 1994](#STD-Aktogu-1994); [Bhalla 2006](#STD-Bhalla-2006); [Bell 1988](#STD-Bell-1988); [Bianchi Porro 1988](#STD-Bianchi-Porro-1988); [Cole 1983](#STD-Cole-1983); [Córdova 1992](#STD-C_x00f3_rdova-1992); [Gilvarry 1990](#STD-Gilvarry-1990); [Korttila 1985](#STD-Korttila-1985); [Lavies 1988](#STD-Lavies-1988); [Lee 1989](#STD-Lee-1989); [Mignonsin 1994](#STD-Mignonsin-1994); [Sainpy 1984](#STD-Sainpy-1984); [Takrouri 1988](#STD-Takrouri-1988); [Tolia 1990](#STD-Tolia-1990); [Whitwam 1983](#STD-Whitwam-1983)); intravenous etomidate in one trial with 17 participants ([Coll-Vinent 2003](#STD-Coll_x002d_Vinent-2003)); intravenous fentanyl in one trial with 126 participants ([Lazaraki 2007](#STD-Lazaraki-2007)); intravenous flunitrazepam in one trial with 86 participants ([Takrouri 1988](#STD-Takrouri-1988)); intravenous placebo in six trials with 548 participants ([Bhalla 2006](#STD-Bhalla-2006); [Eisapour 2015](#STD-Eisapour-2015); [Fakheri 2010](#STD-Fakheri-2010); [Lavies 1988](#STD-Lavies-1988); [Rolo 2012](#STD-Rolo-2012); [Yuno 1996](#STD-Yuno-1996)); intravenous propofol in one trial with 17 participants ([Coll-Vinent 2003](#STD-Coll_x002d_Vinent-2003)); intravenous pethidine hydrochloride in one trial with 120 participants ([Yamasaki 2017](#STD-Yamasaki-2017)); oral chloral hydrate in five trials with 336 participants ([Akil 2005](#STD-Akil-2005); [D'Agostino 2000](#STD-D_x0027_Agostino-2000); [Derakhshanfar 2013](#STD-Derakhshanfar-2013); [Salehi 2017](#STD-Salehi-2017); [Wheeler 2001](#STD-Wheeler-2001)); oral diazepam in two trials with 122 participants ([De Alencar 2010](#STD-De-Alencar-2010); [Everitt 2002](#STD-Everitt-2002)); oral diazepam and clonidine in one trial with 34 participants ([De Alencar 2010](#STD-De-Alencar-2010)); oral ketamine in one trial with 59 participants ([Younge 2001](#STD-Younge-2001)); oral placebo in four trials with 436 participants ([Akil 2005](#STD-Akil-2005); [Kuganeswaran 1999](#STD-Kuganeswaran-1999); [Puttapitakpong 2015](#STD-Puttapitakpong-2015); [Templeton 2010](#STD-Templeton-2010)); intranasal placebo in two trials with 149 participants ([Hollenhorst 2001](#STD-Hollenhorst-2001); [Stokland 2003](#STD-Stokland-2003)); intranasal dexmedetomidine in one trial with 38 participants ([Neville 2016](#STD-Neville-2016)) and intranasal ketamine in one trial with 145 participants ([Alp 2019](#STD-Alp-2019)).

For three trials, we were able to extract data for two different comparisons, as the trials compared midazolam with both a placebo and with chloral hydrate ([Akil 2005](#STD-Akil-2005)) or both placebo and diazepam ([Bhalla 2006](#STD-Bhalla-2006); [Lavies 1988](#STD-Lavies-1988)). Three trials compared midazolam with two different medications ([Coll-Vinent 2003](#STD-Coll_x002d_Vinent-2003); [De Alencar 2010](#STD-De-Alencar-2010); [Takrouri 1988](#STD-Takrouri-1988)). One trial compared two different doses of midazolam with another medication ([Korttila 1985](#STD-Korttila-1985)). For this review, we considered only the outcomes reported from the higher dose of midazolam used, as this dose was comparable with the doses used in the other included trials. Two included articles presented results from the same trial, so we treated this as data from the same study ([Bhalla 2006](#STD-Bhalla-2006)).

#### Financial sources

Financial support was provided by industry for two trials ([Cole 1983](#STD-Cole-1983); [Kuganeswaran 1999](#STD-Kuganeswaran-1999)). Six trials reported receiving funding for their trials from non-industry sources ([Neville 2016](#STD-Neville-2016); [Puttapitakpong 2015](#STD-Puttapitakpong-2015); [Salehi 2017](#STD-Salehi-2017); [Templeton 2010](#STD-Templeton-2010); [Yamasaki 2017](#STD-Yamasaki-2017); [Yuno 1996](#STD-Yuno-1996)). No other trials reported the source of funding in their publications.

### Excluded studies

We needed to review a large number of papers in full text, as it was unclear from the title and abstract whether or not analgesia was administered concurrently with midazolam, and whether or not the sedation was administered intraprocedurally or just before the procedure. We excluded 147 articles that we reviewed in full text. A selection of 31 of these excluded articles is included in the [Characteristics of excluded studies](#CHARACTERISTICS_OF_EXCLUDED_STUDIES) table, to display the common reasons for exclusion. These include intraprocedural sedation used in addition to preprocedural sedation ([Abdel Ghaffar 2018](#STD-Abdel-Ghaffar-2018); [Demiraran 2007](#STD-Demiraran-2007); [Liao 2012](#STD-Liao-2012); [Mishina 2018](#STD-Mishina-2018); [Mui 2005](#STD-Mui-2005); [Muttu 2005](#STD-Muttu-2005); [Onarap 2018](#STD-Onarap-2018)), the concomitant use of analgesia or other sedative medication with midazolam ([Alhashemi 2006](#STD-Alhashemi-2006); [Brouillette 1989](#STD-Brouillette-1989); [Dere 2010](#STD-Dere-2010); [Mercado Longora 2017](#STD-Mercado-Longora-2017); [Nascimento 2007](#STD-Nascimento-2007); [Ominami 2018](#STD-Ominami-2018); [Sajedi 2006](#STD-Sajedi-2006); [Salmon 1992](#STD-Salmon-1992); [Sherry 1989](#STD-Sherry-1989); [Tamayo 1993](#STD-Tamayo-1993); [Wu 2014](#STD-Wu-2014)), routine use of flumazenil ([Ristikankare 1999](#STD-Ristikankare-1999); [Ristikankare 2000a](#STD-Ristikankare-2000a); [Ristikankare 2000b](#STD-Ristikankare-2000b); [Uygur-Bayramiçli 2002](#STD-Uygur_x002d_Bayrami_x00e7_li-2002)), wrong research design ([Frisancho 1996](#STD-Frisancho-1996); [Sandler 1992](#STD-Sandler-1992); [Tesoro 2007](#STD-Tesoro-2007); [Vlastra 2018](#STD-Vlastra-2018); [Weinstein 2010](#STD-Weinstein-2010)), midazolam used in control group if initial sedation was ineffective ([Bonta 2003](#STD-Bonta-2003)), placebo being administered by a different route to midazolam ([Yildirim 2006](#STD-Yildirim-2006)), or the article was a duplicate presentation at a conference ([Derakhshanfar 2017a](#STD-Derakhshanfar-2017a); [Derakhshanfar 2017b](#STD-Derakhshanfar-2017b)). Additionally, in this update, we decided to exclude an additional 6 studies published more than 15 years ago in which we could either not find contact details for clarifications or organise translation (Bardhan 1984; Green 1984;  Ogden 1993; Thakur 2003; Mendes 1986; Wild 1988) on the basis that, considering the duration of time elapsed since publication, it is unlikely these studies will add anything meaningful to the review.

#### Studies awaiting classification

There are no studies awaiting classification.

#### Ongoing studies

There are no ongoing studies identified.

## Risk of bias in included studies

We present summaries of the judgements of the risk of bias of included trials in [Figure 2](#FIG-02) and [Figure 3](#FIG-03). Details of the included trials are in the [Characteristics of included studies](#CHARACTERISTICS_OF_INCLUDED_STUDIES) tables. The overall risk of performance bias and detection bias was low for 50% of the included trials. For randomization sequence generation and allocation concealment, the quality assessment yielded low risk of bias for approximately 25% or less of the included trials. The risk of attrition bias for the primary outcomes was low for more than 75% of trials.

### Allocation (selection bias)

Reporting of methods used for randomization sequence generation and allocation concealment was unclear in the majority of trials. As such, it is unclear as to the impact that potential selection bias might have on the estimates of the effects.

### Blinding (performance bias and detection bias)

Overall, there was a low risk of bias from blinding, due to the double-blinded design used for most trials.

### Incomplete outcome data (attrition bias)

The trials were generally of short duration in an environment that was conducive to a low attrition rate for intra- and post-procedural data that were collected before the participant was discharged. As such, there is low risk of attrition bias for the primary outcomes set for this review. However, one trial ([Everitt 2002](#STD-Everitt-2002)), reported high attrition rates for the 'quality of recovery' outcome, which was measured with a post-discharge survey, meaning there is a high risk of attrition bias, but only for this secondary outcome.

### Selective reporting (reporting bias)

We found no definite evidence of selective reporting. However, we did not seek trial protocols because most included trials were published prior to the establishment of clinical trial registries. It is therefore unclear whether the outcomes infrequently reported or absent from the included trials, such as allergic or anaphylactoid reactions (as defined/measured by the authors of the trial) and sedation reversal, were collected but not reported.

### Other potential sources of bias

We did not identify any other definite source of potential bias.

## Effects of interventions

The results for each comparison included in this review are outlined below. Comparisons were classified as a specific type of sedative medication administered via a specific route. This section is organized as follows. First, the primary comparisons are presented ([Summary of findings table 1](#SOF-01); [Summary of findings table 2](#SOF-02); [Summary of findings table 3](#SOF-03)) followed by the other comparisons in alphabetical order.

### Primary Comparisons

### Intravenous midazolam versus intravenous placebo

Intravenous midazolam was compared with placebo in six trials with 633 adult participants ([Bhalla 2006](#STD-Bhalla-2006); [Fakheri 2010](#STD-Fakheri-2010); [Lavies 1988](#STD-Lavies-1988); [Manning 2016](#STD-Manning-2016); [Rolo 2012](#STD-Rolo-2012); [Yuno 1996](#STD-Yuno-1996)) and one trial with 32 children ([Eisapour 2015](#STD-Eisapour-2015)). The doses of midazolam used are presented in [Table 1](#TBL-01). We downgraded the evidence to low quality on all four primary outcomes, due to concerns about the risk of bias and imprecision ([Summary of findings table 1](#SOF-01)).

#### Primary outcomes

##### Level of sedation on a sedation assessment scale

One study, which used the Ramsay scale to measure level of sedation, reported on this outcome ([Rolo 2012](#STD-Rolo-2012)). Scale scores range from 1 to 6, with higher scores indicating the participant was more sedated.  Participants randomized to midazolam were more sedated (MD 1.05; 95% CI 0.6 to 1.4; 1 study; 100 participants; low-quality; [Analysis 1.1](#CMP-001.01)). The quality of this evidence was downgraded to low quality due to concerns about risk of bias and imprecision.

##### Numeric rating scale of anxiety or number of participants rated as anxious

Two trials ([Rolo 2012](#STD-Rolo-2012) and [Manning 2016](#STD-Manning-2016)) reported on this outcome. There was no difference in anxiety (risk ratio (RR) 0.43; 95% confidence interval (CI) 0.09 to 1.99; I^2^ = 75%; 123 adults; 2 studies; low-quality; [Analysis 1.2](#CMP-001.02)). The quality of this evidence was downgraded to low quality due to concerns about risk of bias and imprecision.

##### Proportion of incomplete procedures or where there was difficulty performing the procedures

Risk of difficulty performing procedures was lower in the midazolam group (RR 0.5; 95% CI 0.29 to 0.86; I^2^ = 45%; 3 studies; 191 adults and 32 children; low-quality; [Analysis 1.3](#CMP-001.03)). The quality of this evidence was downgraded to low quality due to concerns about risk of bias and imprecision.

##### Discomfort/pain

There was no difference in discomfort between groups (RR 0.51; 95% CI 0.25 to 1.04; I^2^ = 0%; 2 studies; 190 participants; low-quality; [Analysis 1.4](#CMP-001.04)). The quality of this evidence was downgraded to low quality due to concerns about risk of bias and imprecision.

#### Secondary outcomes

No trials reported results for disinhibition or excitation, quality of recovery, allergy or anaphylactoid reactions and tolerance of procedure or patient co-operation.

##### Anterograde amnesia (defined by the number of participants who recalled the procedure)

One trial reported this outcome ([Manning 2016](#STD-Manning-2016)). There was no difference between groups (RR 0.83; 95% CI 0.52 to 1.32; 1 study; 23 participants; low-quality evidence).

##### Sedation reversal

[Rolo 2012](#STD-Rolo-2012) (100 participants) reported that no participants required sedation reversal in either group.

##### Participant or proceduralist satisfaction

Four trials, all conducted with adult participants, reported on participant or proceduralist satisfaction ([Bhalla 2006](#STD-Bhalla-2006); [Manning 2016](#STD-Manning-2016); [Rolo 2012](#STD-Rolo-2012); [Yuno 1996](#STD-Yuno-1996)). Midazolam increased the number of participants who were satisfied with sedation (RR 1.21; 95% CI 1.07 to 1.36;  trials = 2; participants = 123; I^2^ = 0%; moderate-quality; [Analysis 1.6](#CMP-001.06)). In the [Yuno 1996](#STD-Yuno-1996) trial, participant satisfaction, which was measured on a four-point scale with lower scores indicating greater satisfaction, was better in the midazolam group (MD -1.65; 95% CI -1.75 to -1.55; 40 participants; moderate-quality; [Analysis 1.7](#CMP-001.07)). Proceduralist satisfaction was also greater in the midazolam group in the same study (MD -1.8; 95% CI -1.9 to -1.7; 1 study; 40 participants; moderate-quality; [Analysis 1.8](#CMP-001.08)). The effect estimates for this outcome are uncertain due to concerns about the risk of bias.

### Oral midazolam versus chloral hydrate

Five trials ([Akil 2005](#STD-Akil-2005); [D'Agostino 2000](#STD-D_x0027_Agostino-2000); [Derakhshanfar 2013](#STD-Derakhshanfar-2013); [Salehi 2017](#STD-Salehi-2017); [Wheeler 2001](#STD-Wheeler-2001)), with 336 participants compared oral midazolam with chloral hydrate for sedation of children ([Summary of findings table 2](#SOF-02)). Doses for midazolam and chloral hydrate differed between the trials ([Table 2](#TBL-02)). A higher dose of midazolam (2mg/kg) was used in the [Salehi 2017](#STD-Salehi-2017) trial compared to the others in this comparison.

#### Primary outcomes

##### Level of sedation on a sedation assessment scale

Two trials reported on the rate of reaching a level of moderate sedation ([Derakhshanfar 2013](#STD-Derakhshanfar-2013); [Salehi 2017](#STD-Salehi-2017)). [Derakhshanfar 2013](#STD-Derakhshanfar-2013) reported the number of patients reaching moderate sedation on Wheeler's sedation scale, and [Salehi 2017](#STD-Salehi-2017) reported the number of patients reaching moderate sedation on the RASS scale. Different scales were used to measure the level of sedation in these studies. [Derakhshanfar 2013](#STD-Derakhshanfar-2013) used Wheeler's sedation level with scores ranging from 1 = agitated to 4 = eyes closing spontaneously but with a response to minor stimuli. [Salehi 2017](#STD-Salehi-2017) reported using the RASS, with the levels of 'alert and calm', 'drowsy', 'light sedation' and 'moderate sedation'. Based on guidelines from the American Society of Anesthesiology, the category in the Wheeler scale that corresponds most closely to 'moderate sedation' was level 4 (eyes closing spontaneously but with a response to minor stimuli) ([American Society of Anesthesiologists 2014](#REF-American-Society-of-Anesthesiologis)). We used this definition for the meta-analysis to combine results from the two studies. Meta-analysis of results suggested that midazolam was less likely to result in moderate sedation compared with chloral (RR 0.30; 95% CI 0.11 to 0.82; I^2^ = 64%; 2 studies; 228 participants; very low-quality; [Analysis 2.1](#CMP-002.01)). We downgraded the evidence from this meta-analysis to very low quality, due to concerns about the risk of bias, inconsistency and imprecision.

##### Numeric rating scale of anxiety or number of participants rated as anxious

A numerical rating of anxiety was reported in two trials with 88 participants. The outcome was measured using different scales (by children using a numerical rating scale in [D'Agostino 2000](#STD-D_x0027_Agostino-2000), and by parents using the Spielberger's Trait Anxiety Inventory in [Akil 2005](#STD-Akil-2005)). The standardized mean difference in anxiety rating was not different (SMD -0.26; 95% CI -0.75 to 0.23; I^2^ = 0%; 2 studies; 68 participants;  low-quality; [Analysis 2.2](#CMP-002.02)). We downgraded the evidence for this outcome to low, due to concerns about the risk of bias and imprecision. To aid interpretation, we converted the estimate for the SMD to an MD using the numerical rating scale in [D'Agostino 2000](#STD-D_x0027_Agostino-2000). Scores ranged from 1 - 5 with lower scores indicating less anxiety)​​​. The standard deviation for the placebo group in this study was 2.97. The mean difference for the meta-analysis was -0.7 (95% CI -2.2 to 0.7).

##### Proportion of incomplete procedures or where there was difficulty performing the procedures

Four trials (268 participants) reported on this outcome ([Akil 2005](#STD-Akil-2005); [D'Agostino 2000](#STD-D_x0027_Agostino-2000); [Derakhshanfar 2013](#STD-Derakhshanfar-2013); [Wheeler 2001](#STD-Wheeler-2001)). Incomplete procedures were more likely in the midazolam group (RR 4.01; 95% CI 1.92 to 8.40; I² = 0%; 4 studies; 436 participants; moderate-quality; [Analysis 2.3](#CMP-002.03)). We downgraded the quality of evidence to moderate, due to concerns about the risk of bias.

##### Discomfort/pain

No trials reported this outcome.

#### Secondary outcomes

Within this comparison, no trials reported results for anterograde amnesia, quality of recovery, allergic or anaphylactoid reactions, sedation reversal, and patient or proceduralist satisfaction.

##### Disinhibition or excitation

There was no difference in disinhibition or excitation between midazolam or chloral hydrate groups in [Derakhshanfar 2013](#STD-Derakhshanfar-2013) (RR 1.0; 95% CI 0.39 to 2.55; 1 study; 160 participants; [Analysis 2.4](#CMP-002.04)). No events were observed in either group in [Wheeler 2001](#STD-Wheeler-2001) (40 participants). Quality of evidence was downgraded to low quality due to concerns about risk of bias and imprecision.

##### Tolerance of procedure or participant co-operation

Tolerance of the procedure was measured using the Frankl behaviour rating scale (range 1 to 4, with higher scores indicating better tolerance) in [Akil 2005](#STD-Akil-2005). There was no difference in tolerance between groups (MD 0.25; 95% CI -0.9 to 0.4; 1 study; 35 participants; low-quality; [Analysis 2.5](#CMP-002.05)). Participant co-operation was measured using the Houpt behavioural scale (range 1 to 6, with higher scores indicating better co-operation) in the [Akil 2005](#STD-Akil-2005) trial and there was no difference between groups (MD 0.16; 95% CI -0.54 to 0.86; 1 study; 35 participants; low-quality; [Analysis 2.6](#CMP-002.06)). The evidence for this outcome was rated as low quality due to concerns about risk of bias and imprecision.

### Oral midazolam versus placebo

Four trials ([Akil 2005](#STD-Akil-2005); [Kuganeswaran 1999](#STD-Kuganeswaran-1999); [Puttapitakpong 2015](#STD-Puttapitakpong-2015); [Templeton 2010](#STD-Templeton-2010)) with 436 participants compared midazolam administered via the oral route with a placebo ([Summary of findings table 3](#SOF-03)). Two trials were conducted with adults and two with children. [Kuganeswaran 1999](#STD-Kuganeswaran-1999) was conducted in adults undergoing outpatient sigmoidoscopy (99 participants). [Puttapitakpong 2015](#STD-Puttapitakpong-2015) was conducted with 260 adult participants aged 18 to 70 undergoing elective oesophagogastroduodenoscopy. [Templeton 2010](#STD-Templeton-2010) was conducted in children undergoing removal of Kirschner wires (42 participants). [Akil 2005](#STD-Akil-2005) enrolled children undergoing micturating cystourethrography (35 participants).

#### Primary outcomes

##### Level of sedation on a sedation assessment scale

[Kuganeswaran 1999](#STD-Kuganeswaran-1999) reported on level of sedation measured on a 4-point scale with higher scores indicating a greater sedative effect. Although it was reported that level of sedation was measured every 5 minutes, summary statistics were reported only for the timepoint 10 minutes after administration of midazolam. At this timepoint, sedation level was higher in the midazolam group (MD 0.2; 95% CI 0.19 to 0.21; 101 participants; low-quality evidence; [Analysis 3.3](#CMP-003.01)).

##### Numeric rating scale of anxiety or number of participants rated as anxious

A numerical rating of anxiety was reported in all trials included in this comparison. Standardized mean difference was used for meta-analysis because different scales were used in each trial. Midazolam reduced ratings of anxiety by one standard deviation (SMD -1.01; 95% CI -1.86 to -0.16; I^2^ = 92%; 4 studies; 436 participants; low quality; [Analysis 3.2](#CMP-003.02)). The quality of this evidence was downgraded to low quality due to concerns about the risk of bias and imprecision. To aid interpretation, we converted the estimate for the SMD to an MD using the numerical rating scale in [Puttapitakpong 2015](#STD-Puttapitakpong-2015). Scores ranged from 0 to 10, with lower scores indicating less anxiety)​​​. The standard deviation for the placebo group in this study was 1.9. The mean difference for the meta-analysis was -1.9 (95% CI = -3.5  to 0.3 ).

##### Proportion of incomplete procedures or where there was difficulty performing the procedures

There were no incomplete procedures in either the midazolam or placebo groups in the [Kuganeswaran 1999](#STD-Kuganeswaran-1999), [Puttapitakpong 2015](#STD-Puttapitakpong-2015) and [Templeton 2010](#STD-Templeton-2010) trials. One procedure (6%) could not be completed in the midazolam group in [Akil 2005](#STD-Akil-2005).

##### Discomfort/pain

In the [Kuganeswaran 1999](#STD-Kuganeswaran-1999) trial, which was conducted with adult participants undergoing sigmoidoscopy, pain was lower in the midazolam group (MD -2; 95% CI -2.5 to -1.6; 1 study; 99 participants; moderate-quality; [Analysis 3.3](#CMP-003.03)). Quality of evidence was downgraded due to concerns about the risk of bias.

#### Secondary outcomes

Within this comparison, no trials reported results for disinhibition or excitation, quality of recovery, allergic or anaphylactoid reactions and sedation reversal.

##### Anterograde amnesia (defined by number of participants who recalled the procedure)

There was no overall difference in anterograde amnesia between midazolam and placebo in meta-analysis of two trials that enrolled adults undergoing upper ([Puttapitakpong 2015](#STD-Puttapitakpong-2015)) or lower ([Kuganeswaran 1999](#STD-Kuganeswaran-1999)) endoscopy (RR 0.32, 95% CI 0.01 to 10.12; I^2^ = 99%; 2 trials; 359 participants; low-quality;  [Analysis 3.4](#CMP-003.04)). However, the results were inconsistent and imprecise. As such, the quality of evidence was rated as low quality.

##### Tolerance of procedure or participant co-operation

Tolerance of the procedure was measured using the Frankl behaviour rating scale (range 1 to 4, with higher scores indicating better tolerance) in [Akil 2005](#STD-Akil-2005). There was no difference in tolerance between groups (MD -0.13, 95% CI -0.5 to 0.76; 1 study; 35 participants; low-quality; [Analysis 3.5](#CMP-003.05)). This effect estimate is uncertain due to concerns about imprecision and the risk of bias. Tolerance of the procedure (defined as not willing to repeat the procedure with the same sedation) was better in the midazolam group in the [Puttapitakpong 2015](#STD-Puttapitakpong-2015) trial. Fewer participants in the midazolam group were not willing to repeat the procedure with the same sedation (RR 0.1 95% CI 0.01 to 0.77; 1 study; 260 participants; low-quality; [Analysis 3.6](#CMP-003.06)). This effect estimate is uncertain due to concerns about imprecision and the risk of bias.

Participant co-operation was measured using the Houpt behavioural scale (range 1 to 6, with higher scores indicating better co-operation) in [Akil 2005](#STD-Akil-2005). Participant co-operation between groups was higher in the midazolam group, but the effect estimate was imprecise, and there were concerns about the risk of bias (MD 0.82, 95% CI 0.1 to 1.54; 1 study; 35 participants; low-quality; [Analysis 3.7](#CMP-003.07)).

##### Participant or proceduralist satisfaction

Participant satisfaction (measured by participants' perception that they received inadequate sedation for their procedure) in [Kuganeswaran 1999](#STD-Kuganeswaran-1999), was superior in the midazolam group (RR 0.43 95% CI 0.26 to 0.7; 1 study; 99 participants; low-quality; [Analysis 3.9](#CMP-003.09)). This effect estimate is uncertain due to concerns about imprecision and the risk of bias. In the [Puttapitakpong 2015](#STD-Puttapitakpong-2015) trial, ratings of satisfaction on a scale from 0-10 (higher scores = greater satisfaction) from participants (MD 2.5, 95% CI 2.18 to 2.82; 1 study; 260 participants; moderate-quality; [Analysis 3.8](#CMP-003.08)) and proceduralists (MD 2.3 95% CI 2.02 to 2.58; 1 study; 260 participants; moderate-quality due to concerns about the risk of bias; [Analysis 3.10](#CMP-003.10)) were higher in the midazolam group. The effect estimates from this trial are uncertain due to concerns about the risk of bias.

### Secondary Comparisons

### Intravenous midazolam versus diazepam

Intravenous midazolam was compared with diazepam in 15 trials with 1339 participants ([Aktogu 1994](#STD-Aktogu-1994); [Bhalla 2006](#STD-Bhalla-2006); [Bell 1988](#STD-Bell-1988); [Bianchi Porro 1988](#STD-Bianchi-Porro-1988); [Cole 1983](#STD-Cole-1983); [Córdova 1992](#STD-C_x00f3_rdova-1992); [Gilvarry 1990](#STD-Gilvarry-1990); [Korttila 1985](#STD-Korttila-1985); [Lavies 1988](#STD-Lavies-1988); [Lee 1989](#STD-Lee-1989); [Mignonsin 1994](#STD-Mignonsin-1994); [Sainpy 1984](#STD-Sainpy-1984); [Takrouri 1988](#STD-Takrouri-1988); [Tolia 1990](#STD-Tolia-1990); [Whitwam 1983](#STD-Whitwam-1983)). We present the doses of midazolam and diazepam that were used in each of these trials in [Table 3](#TBL-03).

#### Primary outcomes

##### Level of sedation on a sedation assessment scale

Two trials with 175 participants measured the level of sedation, but each reported this outcome differently. One trial with 75 participants ([Takrouri 1988](#STD-Takrouri-1988)) reported on the difference in the level of sedation on a sedation assessment scale. The mean level of sedation was 0.5 higher (on a scale that ranged from 0 to 4) in the midazolam group. No effect estimate or measures of variance were reported in the trial. More participants who received midazolam were deemed to be in a satisfactory sedation state in the [Mignonsin 1994](#STD-Mignonsin-1994) trial, but the effect estimate is imprecise (RR 2.3; 95% CI 1.03 to 5.3; 1 study; 100 participants; low-quality evidence; [Analysis 4.11](#CMP-004.01)).

##### Numeric rating scale of anxiety or number of participants rated as anxious

Two trials (175 participants) reported on this outcome ([Takrouri 1988](#STD-Takrouri-1988); [Whitwam 1983](#STD-Whitwam-1983)). There was no difference in the number of participants who were rated as anxious (RR 0.80, 95% CI 0.39 to 1.62; I² = 0%; low-quality;  [Analysis 4.1](#CMP-004.02)). This effect estimate is uncertain due to concerns about imprecision and the risk of bias.

##### Proportion of incomplete procedures or where there was difficulty performing the procedures

Meta-analysis of results from three trials ([Lee 1989](#STD-Lee-1989); [Takrouri 1988](#STD-Takrouri-1988); [Whitwam 1983](#STD-Whitwam-1983)), for the number of procedures rated as 'difficult to perform' revealed no important difference between the midazolam group (RR 0.66, 95% CI 0.41 to 1.07; I^2^ = 0%; 3 studies; 277 participants; low-quality; [Analysis 4.2](#CMP-004.03)). This effect estimate is uncertain due to concerns about imprecision and the risk of bias. One trial with 170 participants was not included in the meta-analysis because there were no incomplete procedures in either group ([Bhalla 2006](#STD-Bhalla-2006)).

##### Discomfort/pain

In a meta-analysis of six trials ([Cole 1983](#STD-Cole-1983); [Lee 1989](#STD-Lee-1989); [Bhalla 2006](#STD-Bhalla-2006); [Mignonsin 1994](#STD-Mignonsin-1994); [Sainpy 1984](#STD-Sainpy-1984); [Tolia 1990](#STD-Tolia-1990)), participants who received midazolam reported less discomfort or pain than those who received diazepam (RR 0.44, 95% CI 0.17 to 1.15; I² = 78%; 6 studies; 515 participants; low-quality; [Analysis 4.3](#CMP-004.04)). This effect estimate is uncertain due to concerns about imprecision and the risk of bias.

#### Secondary outcomes

Within this comparison, no trials reported results for allergic or anaphylactoid reactions and sedation reversal.

##### Anterograde amnesia (defined by number of participants who recalled the procedure)

A meta-analysis of results from ten trials ([Aktogu 1994](#STD-Aktogu-1994); [Córdova 1992](#STD-C_x00f3_rdova-1992); [Gilvarry 1990](#STD-Gilvarry-1990); [Korttila 1985](#STD-Korttila-1985); [Lee 1989](#STD-Lee-1989); [Mignonsin 1994](#STD-Mignonsin-1994); [Sainpy 1984](#STD-Sainpy-1984); [Takrouri 1988](#STD-Takrouri-1988); [Tolia 1990](#STD-Tolia-1990); [Whitwam 1983](#STD-Whitwam-1983)) with 687 participants revealed that those who received midazolam were more likely to experience anterograde amnesia than those who received diazepam (RR 0.55, 95% CI 0.33 to 0.94; 10 studies; 687 participants; very low-quality evidence due to concerns about the risk of bias and inconsistency; I² = 72%; [Analysis 4.5](#CMP-004.05)). This effect estimate is highly uncertain due to concerns about imprecision, inconsistency and the risk of bias. It should be noted that two participants randomized to the midazolam group in [Tolia 1990](#STD-Tolia-1990) withdrew, which meant that there were missing data for this outcome. Sensitivity analysis using best-case and worst-case scenarios did not significantly change the result ([Analysis 4.9](#CMP-004.06); [Analysis 4.10](#CMP-004.07)).

##### Disinhibition or excitation

More participants in the midazolam group experienced disinhibition or excitation in the [Mignonsin 1994](#STD-Mignonsin-1994) trial (RR=1.28; 95% CI=1.00 to 1.64; 1 study; 100 participants; low-quality evidence; [Analysis 4.12](#CMP-004.08)). This effect estimate is uncertain due to concerns about imprecision and the risk of bias.

##### Quality of recovery

Three trials ([Cole 1983](#STD-Cole-1983); [Korttila 1985](#STD-Korttila-1985); [Takrouri 1988](#STD-Takrouri-1988)) with 166 participants reported on quality of recovery (measured in the trials as delayed recovery). There was no difference between groups (RR 0.72; 95% CI 0.08 to 6.63; I^2^ = 67%; 3 studies; 166 participants; low-quality; [Analysis 4.4](#CMP-004.09)). This effect estimate is uncertain due to concerns about imprecision and inconsistency.

##### Tolerance of procedure or participant co-operation

Five trials ([Lee 1989](#STD-Lee-1989); [Bhalla 2006](#STD-Bhalla-2006); [Takrouri 1988](#STD-Takrouri-1988); [Tolia 1990](#STD-Tolia-1990); [Whitwam 1983](#STD-Whitwam-1983)), including 486 participants reported on this outcome. There was no difference between groups, but the quality of evidence was very low due to concerns about risk of bias, imprecision and inconsistency (RR 0.93, 95% CI 0.51 to 1.68; I^2^ = 62%; 5 studies; 486 participants; very low-quality; [Analysis 4.6](#CMP-004.10)).

##### Participant or proceduralist satisfaction

Two trials ([Cole 1983](#STD-Cole-1983); [Korttila 1985](#STD-Korttila-1985)), with 91 participants reported proceduralist satisfaction and participant satisfaction using a scale from 0 to 100, with higher scores equating to better satisfaction. Meta-analysis identified that participant satisfaction (MD 2.17, 95% CI -0.51 to 4.85; I^2^ = 12%; 2 studies; 91 participants; moderate-quality; [Analysis 4.8](#CMP-004.12)) and proceduralist satisfaction (MD 1.09, 95% CI -10.43 to 12.60; 2 studies; 91 participants; moderate-quality; ​​​​​​ [Analysis 4.7](#CMP-004.11)) was no different between groups. The quality of evidence was rated as moderate due to concerns about risk of bias.

### Intravenous midazolam versus etomidate

We identified one trial with 17 participants that investigated the use of midazolam versus etomidate before electrical cardioversion ([Coll-Vinent 2003](#STD-Coll_x002d_Vinent-2003)). Eight participants were randomized to midazolam and nine to receive etomidate. The doses of both midazolam and etomidate were 0.2 mg/kg. This trial did not report on the outcomes included in this review.

### Intravenous midazolam versus fentanyl

Intravenous midazolam was compared with fentanyl for sedation before colonoscopy in one trial with 126 participants ([Lazaraki 2007](#STD-Lazaraki-2007)). Mean dosage for midazolam was 4.6 mg and for fentanyl was 36 mcg. Within this comparison, no trials reported results for any of the primary outcomes, disinhibition or excitation, quality of recovery, allergic or anaphylactoid reactions and tolerance of procedure or patient co-operation.

#### Secondary outcomes

##### Anterograde amnesia (defined by number of participants who recalled the procedure)

Midazolam produced more anterograde amnesia (RR 0.54; 95% CI 0.42 to 0.68; 1 study; 126 participants; moderate-quality; [Analysis 5.1](#CMP-005.01)). This effect estimate is uncertain due to concerns about the risk of bias.

##### Sedation reversal

There were no differences between groups in the number of participants who required sedation reversal (no events in either group).

##### Participant or proceduralist satisfaction

There were no differences between the groups in the effectiveness of the sedation in terms of participant satisfaction, which was measured in this trial as the acceptability of undergoing another procedure with the same sedative medication (RR 1.38; 95% CI 0.39 to 4.88; 1 study; 126 participants; low quality; [Analysis 5.2](#CMP-005.02)). This effect estimate is uncertain due to concerns about imprecision and the risk of bias.

### Intravenous midazolam versus flunitrazepam

We identified one trial with 86 participants that compared intravenous midazolam with flunitrazepam ([Takrouri 1988](#STD-Takrouri-1988)) before gastrointestinal endoscopy. The mean dose of midazolam was 5.8 mg and 0.65 mg for flunitrazepam. In this trial, 41 participants were randomized to midazolam and 45 participants to flunitrazepam. Within this comparison, no trials reported results for anxiety, discomfort/pain,  disinhibition or excitation, allergic or anaphylactoid reactions, sedation reversal and participant or proceduralist satisfaction.

#### Primary outcomes

##### Level of sedation on a sedation assessment scale

The mean level of sedation was 0.5 higher (on a scale that ranged from 0 to 4) in the midazolam group. No effect estimate or measures of variance were reported in the trial.

##### Proportion of incomplete procedures or where there was difficulty performing the procedures

There were no differences between groups for difficulty performing procedures (RR 1.25; 95% CI 0.5 to 3.15; low quality; [Analysis 6.1](#CMP-006.01)). This effect estimate is uncertain due to concerns about imprecision and the risk of bias.

#### Secondary outcomes

##### Anterograde amnesia (defined by number of participants who recalled the procedure)

The risk of recalling a procedure was reduced in the midazolam group (RR 0.34; 95% CI 0.2 to 0.57; 1 study; 41 participants; moderate-quality; [Analysis 6.2](#CMP-006.02)). This effect estimate is uncertain due to concerns about the risk of bias.

##### Quality of recovery

Delayed recovery was lower in the midazolam group (RR 0.03; 95% CI 0 to 0.5; 1 study; 41 participants; low-quality; [Analysis 6.3](#CMP-006.03)). This effect estimate is uncertain due to concerns about imprecision and the risk of bias.

##### Tolerance of procedure or participant co-operation

There were no differences between groups for participant co-operation, which was measured in this trial as the number of participants who were rated as 'not co-operative' (RR 0.82; 95% CI 0.39 to 1.75; 1 study; 41 participants; low-quality; [Analysis 6.4](#CMP-006.04)). This effect estimate is uncertain due to concerns about imprecision and the risk of bias.

### Intravenous midazolam versus propofol

We identified one trial with 17 participants that investigated the use of midazolam versus propofol before electrical cardioversion ([Coll-Vinent 2003](#STD-Coll_x002d_Vinent-2003)). The midazolam group (eight participants) were given a dose of 0.2 mg/kg, and the propofol group (nine participants) were given a dose of 1.5 mg/kg. Within this comparison, no trials reported results for incomplete or difficulty performing procedures, discomfort/pain, anterograde amnesia, disinhibition or excitation, quality of recovery, allergic or anaphylactoid reactions, sedation reversal, tolerance of procedure or patient co-operation and patient or proceduralist satisfaction.

#### Primary outcomes

##### Level of sedation on a sedation assessment scale

Level of sedation (measured using the Ramsay scale) was similar between groups. The median score was 6, with interquartile range 5 and 6 in both groups.

### Intravenous midazolam versus pethidine hydrochloride

We identified one trial ([Yamasaki 2017](#STD-Yamasaki-2017)) that enrolled 120 participants for this comparison. Forty participants were randomized to midazolam and 39 to pethidine hydrochloride. Participants in the midazolam group received intravenous midazolam in 0.5-1.0mg doses administered until a Ramsay score of 3 was achieved for pharyngeal observation. Participants in the pethidine group received 35mg of intravenous pethidine hydrochloride. Within this comparison, no trials reported results for level of sedation, anxiety, incomplete or difficulty performing procedures, anterograde amnesia, disinhibition or excitation, quality of recovery, allergic or anaphylactoid reactions, sedation reversal, tolerance of procedure or patient co-operation and participant or proceduralist-satisfaction.

#### Primary outcomes

##### Discomfort/pain

[Yamasaki 2017](#STD-Yamasaki-2017) reported on participants' discomfort during pharyngeal observation using a visual analogue scale. Participants rated their level of discomfort between 0 mm - 100 mm along a 100 mm horizontal line, where higher values indicated greater pain. The mean score for discomfort was not significantly different between the midazolam and pethidine groups (MD -0.4; 95% CI -1.39 to 0.59; 1 study; 120 participants; low-quality; [Analysis 7.1](#CMP-007.01)). This effect estimate is uncertain due to concerns about imprecision and the risk of bias.

#### Oral midazolam versus diazepam

Oral midazolam was compared with diazepam in two trials with 122 participants ([De Alencar 2010](#STD-De-Alencar-2010); [Everitt 2002](#STD-Everitt-2002)). In [De Alencar 2010](#STD-De-Alencar-2010), which enrolled adults undergoing office-based plastic surgery, the midazolam group was given a 15 mg dose, and the diazepam group was given a 10 mg dose. The midazolam dose was 1.0 mg/kg (maximum 15 mg), and the diazepam dose was 0.5 mg/kg (maximum 10 mg) in [Everitt 2002](#STD-Everitt-2002), which enrolled children undergoing laceration repair. Within this comparison, no trials reported results for anxiety, incomplete or difficulty performing procedures, discomfort/pain, anterograde amnesia, disinhibition or excitation, allergic or anaphylactoid reactions, sedation reversal, tolerance of procedure or patient co-operation and participant or proceduralist-satisfaction.

#### Primary outcomes

##### Level of sedation on a sedation assessment scale

More participants who received midazolam in [De Alencar 2010](#STD-De-Alencar-2010) were sedated to the level of 'somnolence' (RR 4.94; 95% CI 1.72 to 14; 1 study; 35 participants; [Analysis 8.1](#CMP-008.01)). This effect estimate is uncertain due to concerns about imprecision and the risk of bias. In the [Everitt 2002](#STD-Everitt-2002) trial that compared oral midazolam with diazepam in children undergoing laceration repair, midazolam produced higher levels of sedation (MD -17; 95% CI -28 to 6; 1 study; 87 participants; low-quality; [Analysis 8.2](#CMP-008.02)). This effect estimate is uncertain due to concerns about imprecision and the risk of bias.

#### Secondary outcomes

##### Quality of recovery

Quality of recovery was reported in one trial ([Everitt 2002](#STD-Everitt-2002)). More children were reported to be drowsy after discharge in the midazolam group (RR 1.6; 95% CI 0.87 to 2.91;1 study; 87 participants; low quality; [Analysis 8.3](#CMP-008.03)). This effect estimate is uncertain due to concerns about imprecision and the risk of bias.

### Oral midazolam versus diazepam and clonidine

Oral midazolam was compared with a combination of diazepam and clonidine in one trial with 34 participants ([De Alencar 2010](#STD-De-Alencar-2010)). Seventeen participants were randomized to receive 15 mg midazolam and 17 participants to receive 10 mg diazepam with 0.15 mg clonidine. Within this comparison, no trials reported results for anxiety, incomplete or difficulty performing procedures, discomfort/pain and any of the secondary outcomes.

#### Primary outcomes

##### Level of sedation on a sedation assessment scale

Level of sedation was measured using the Michigan University Scale, with a range in scores from 0 = awake to 4 = unrousable to stimuli. Measures of central tendency were not reported in the article.

### Oral midazolam versus ketamine

[Younge 2001](#STD-Younge-2001) compared 0.7 mg/kg oral midazolam with 10 mg/kg oral ketamine in an RCT for sedation before laceration repair in 59 children. Within this comparison, no trials reported results for anxiety, incomplete or difficulty performing procedures, discomfort/pain, anterograde amnesia, quality of recovery, allergic or anaphylactoid reactions, sedation reversal, tolerance of procedure or patient co-operation and participant or proceduralist satisfaction.

#### Primary outcomes

##### Level of sedation on a sedation assessment scale

Children who received ketamine were more deeply sedated (median score 2 versus 3 (lower score = deeper sedation). The p-value for this difference was reported to be 0.023. We used this value to estimate confidence intervals for a median difference of 1 between groups to be 0.14 to 1.9. We downgraded the evidence to low-quality due to concerns about the risk of bias and imprecision.

#### Secondary outcomes

##### Disinhibition or excitation

Disinhibition/excitation was more likely to occur in the midazolam group (RR 13.43; 95% CI 0.79 to 228; 1 study; 59 participants; low-quality; [Analysis 9.1](#CMP-009.01)). This effect estimate is uncertain due to concerns about imprecision and the risk of bias.

### Intranasal midazolam versus placebo

Two trials (149 participants) compared midazolam administered via the intranasal route for sedation before a procedure with placebo ([Hollenhorst 2001](#STD-Hollenhorst-2001); [Stokland 2003](#STD-Stokland-2003)). [Hollenhorst 2001](#STD-Hollenhorst-2001) compared intranasal midazolam with placebo in adults undergoing magnetic resonance imaging, while [Stokland 2003](#STD-Stokland-2003) was conducted in children requiring cystourethrography. Within this comparison, no trials reported results for discomfort/pain and any of the secondary outcomes.

#### Primary outcomes

##### Level of sedation on a sedation assessment scale

A deeper level of sedation was observed in the midazolam group in [Hollenhorst 2001](#STD-Hollenhorst-2001) (MD 0.59; 95% CI 0.31 to 0.87; 1 study; 54 participants; moderate-quality; [Analysis 10.1](#CMP-010.01)). We downgraded this evidence to moderate quality, due to concerns about the risk of bias arising from an unclear risk of bias from randomization sequence generation and allocation concealment.

##### Numeric rating scale of anxiety or number of participants rated as anxious

[Hollenhorst 2001](#STD-Hollenhorst-2001) reported a marked reduction in a numerical rating of anxiety among participants who received midazolam prior to their magnetic resonance imaging procedure (mean 17.3 (SD 18.58) in midazolam group; mean 49.3 (SD 29.46) in placebo group; P < 0.001) (MD -32; 95% CI -45 to -18; 1 study; 54 participants; low-quality; [Analysis 10.2](#CMP-010.02)). We downgraded this evidence to low quality, due to concerns about the risk of bias arising from an unclear risk of bias from randomization sequence generation and allocation concealment as well as imprecision.

##### Proportion of incomplete procedures or where there was difficulty performing the procedures

One of the primary outcomes, incomplete procedures, was reported in both of these trials ([Hollenhorst 2001](#STD-Hollenhorst-2001); [Stokland 2003](#STD-Stokland-2003)). Meta-analysis of results from the 149 participants showed that the administration of midazolam compared with placebo had no impact on incomplete procedures (RR 0.14, 95% CI 0.02 to 1.12;  I^2^ = 0%;  2 studies; low-quality; 149 participants; [Analysis 10.3](#CMP-010.03)). The evidence was downgraded due to concerns about the risk of bias and imprecision.

### Intranasal midazolam versus dexmedetomidine

One trial ([Neville 2016](#STD-Neville-2016)) with 38 participants compared intranasal midazolam with intranasal dexmedetomidine for sedation in children before laceration repair. Eighteen participants were randomized to receive 0.4mg/kg of intranasal midazolam, and twenty participants received 2mcg/kg of intranasal dexmedetomidine. Within this comparison, no trials reported results for the level of sedation, incomplete or difficulty performing procedures, discomfort/pain and any of the secondary outcomes.

#### Primary outcome

##### Numeric rating scale of anxiety or number of participants rated as anxious

[Neville 2016](#STD-Neville-2016) reported on participants' level of anxiety during patient positioning for the procedure as measured by the modified Yale Preoperative Anxiety Scale. Participants were observed for five categories (activity, vocalizations, emotional expressivity, state of apparent arousal, and use of parents) combined to produce a total anxiety score between 23.3 and 100, where higher values indicated greater anxiety. The dexmedetomidine group had a median anxiety score that was significantly lower compared to the midazolam group (23.3 (IQR 23-35) dexmedetomidine; 36.3 (IQR 33-41) midazolam), with a difference in score of 9.2 points (95% CI 5.0 to 13.3; P = 0.007).  [Neville 2016](#STD-Neville-2016) also reported on the proportion of participants who were not anxious during positioning for the procedure. Participants who scored less than 30 using the modified Yale Preoperative Anxiety Scale were considered "not anxious". More participants in the dexmedetomidine group were not anxious during positioning compared to those in the midazolam group (14/20 (70%) dexmedetomidine; 2/18 (11%) midazolam, P = 0.00). The odds of participants not being anxious during positioning was 19 times higher in the dexmedetomidine group compared to the midazolam group (OR 19, 95% CI 3 to 108). We rated this evidence as moderate quality, due to concerns about imprecision.

#### Secondary outcomes

None of the secondary outcomes for this comparison were reported by the included trials.

### Intranasal midazolam versus ketamine

One trial ([Alp 2019](#STD-Alp-2019)), with 145 children undergoing echocardiography, compared intranasal midazolam with ketamine. There were 73 participants allocated to receive midazolam (0.2mg/kg) and 27 participants to ketamine (4mg/kg). Within this comparison, no trials reported results for anxiety, discomfort/pain and any of the secondary outcomes.

#### Primary outcome

##### Level of sedation

[Alp 2019](#STD-Alp-2019) measured level of sedation every 15 minutes using the RASS, with levels of 'awake and calm', 'drowsy' or 'sedated'. More participants were rated as 'sedated' in the midazolam group at 15 minutes (RR 50; 95% CI 3 to 809; 1 trial; 145 participants; low-quality; [Analysis 11.1](#CMP-011.01)) and 30 minutes (RR 2; 95% CI 1.3 to 3.3; 1 trial; 145 participants; low-quality; [Analysis 11.2](#CMP-011.02)). There was no difference in the level of sedation between groups at 45 minutes (RR 0.97; 95% CI 0.88 to 1.67; 1 trial; 145 participants; low-quality; [Analysis 11.3](#CMP-011.03)) and 60 minutes (RR 1.0; 95% CI 0.97 to 1.03; 1 trial; 145 participants; low-quality; [Analysis 11.4](#CMP-011.04)). The effect estimates for this outcome are uncertain due to concerns about imprecision and the risk of bias.

##### Incomplete procedures

There was no difference between groups in the number of participants who were insufficiently sedated to be able to perform the procedure (RR 0.99; 95% CI 0.21 to 4.73; 1 trial; 145 participants; low quality; [Analysis 11.5](#CMP-011.05)). This effect estimate is uncertain due to concerns about imprecision and the risk of bias.
